# Supplementary material for: Quantitative 3D determination of self-assembled structures on nanoparticles using small angle neutron scattering
Source: Nat Commun. 2018 Apr 9;9:1343. doi: 10.1038/s41467-018-03699-7 (PMC5890256; doi:10.1038/s41467-018-03699-7)
Supplement: Supplementary file 1 — Supplementary Information [file 41467_2018_3699_MOESM1_ESM.pdf]

SUPPLEMENTARY INFORMATION

Quantitative 3D determination of self-assembled structures on nanoparticles using small angle neutron scattering

Zhi Luo, Domenico Marson, Quy K. Ong, Joachim Kohlbrecher, Aurel Radulescu, Anna Loiudice, Anwen Krause-Heuer, Tamim Darwish, Sandor Balog, Raffaella Buonsanti, Dmitri I. Svergun, Paola Posocco & Francesco Stellacci*

Size and ligand ratio characterization of nanoparticles

NMR data of PET-DDT nanoparticles before core etching with iodine

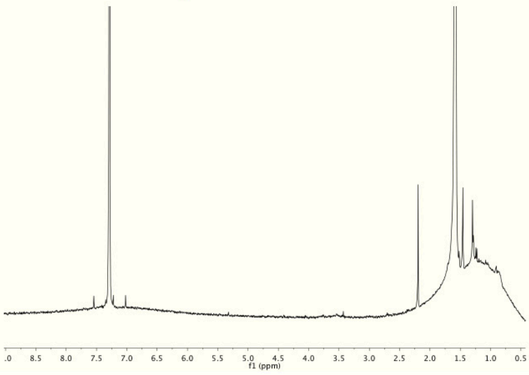

5.5 ± 0.5 nm PET-DDT gold nanoparticles

TEM

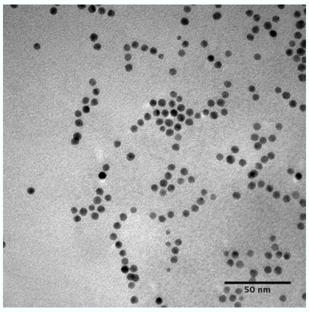

SAXS

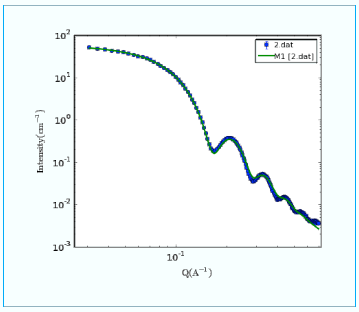

Size distribution

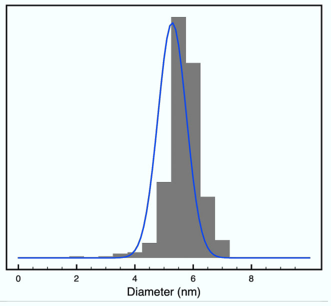

NMR

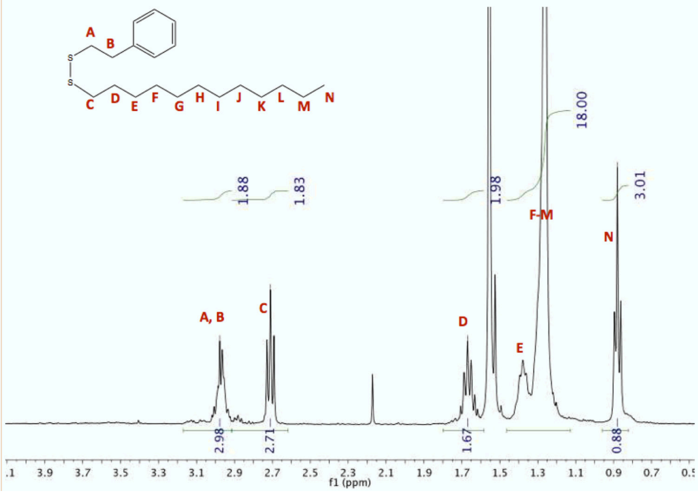

4.4 ± 0.4 nm PET-DDT gold nanoparticles

TEM

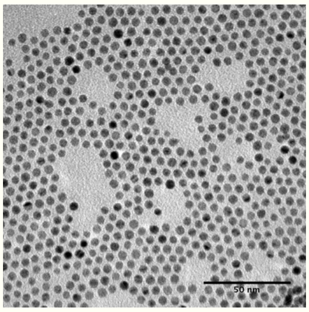

SAXS

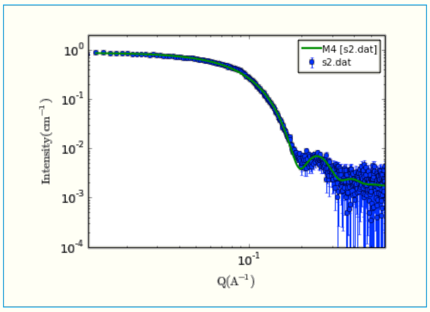

Size distribution

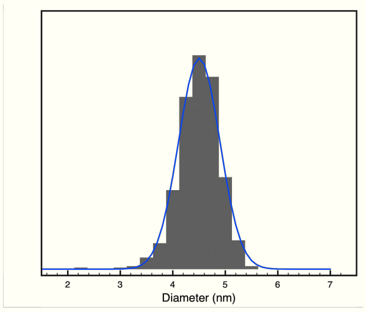

NMR

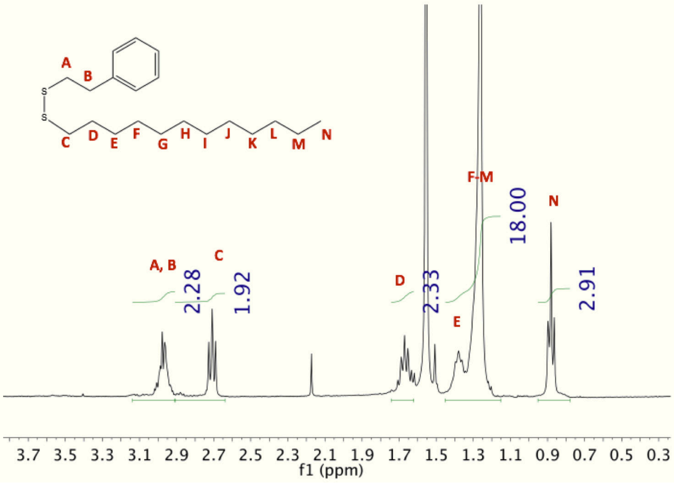

3.2 ± 0.3 nm PET-DDT gold nanoparticles

TEM

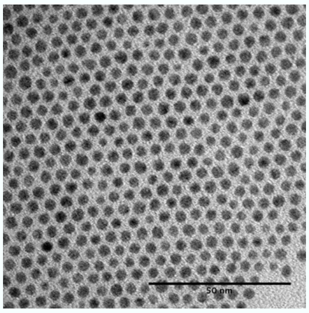

SAXS

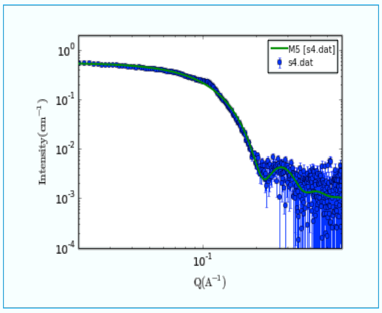

Size distribution

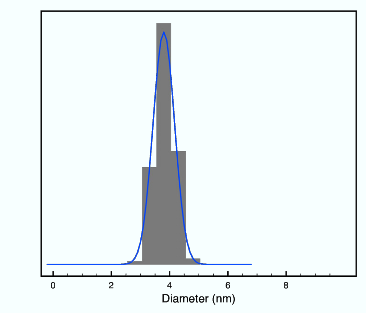

NMR

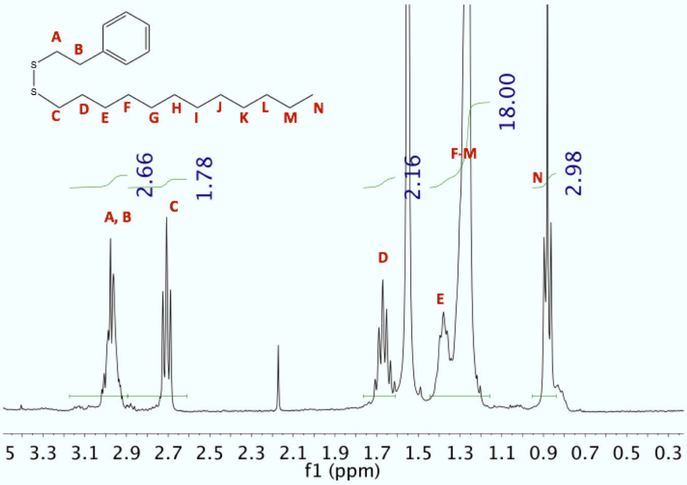

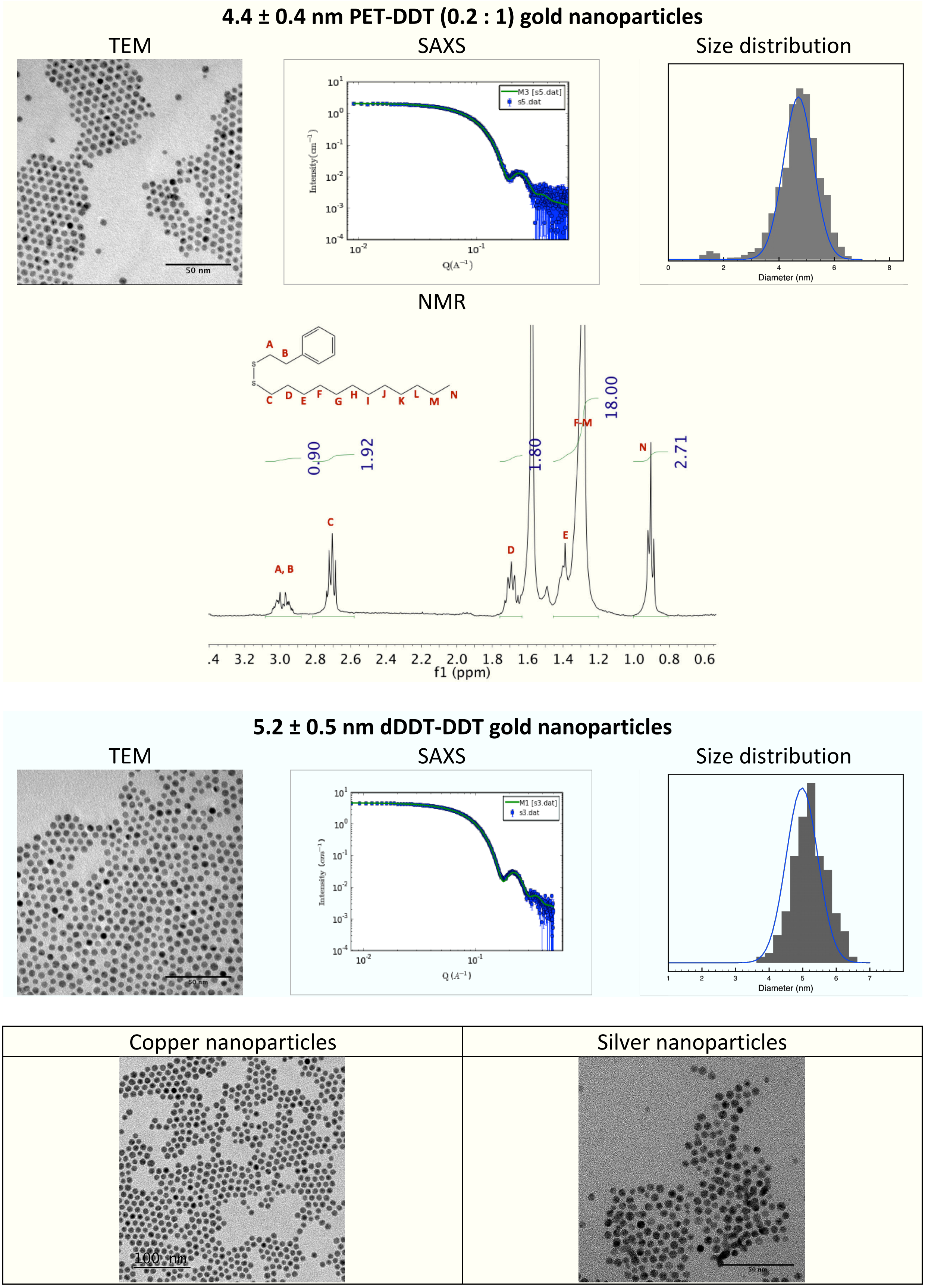

Supplementary Figure 1. Core size (TEM and SAXS) and NMR characterization of the nanoparticles.

Supplementary Note 1. FTIR measurements of PET-DDT nanoparticles

In order to prove that the deuteration does not affect the ligand ratio on the nanoparticles, we used FTIR to measure the PET-DDT and PET-dDDT samples. As shown below, the FTIR spectra of a mixture of DDT and dDDT homoligand nanoparticles were recorded at varying molar ratios of the nanoparticles, Figure S3A. The ratio of the intensity of the CH2 and CD2 stretching peaks was then calculated and plotted against the molar ratio of the nanoparticles in order to build a calibration curve, Figure S3B. Then the FTIR spectra of PET-DDT and PET-dDDT nanoparticles were recorded, Figure S3C. The CD2 peak intensity of the PET-dDDT nanoparticles was then converted to the corresponding CH2 intensity using the calibration curve discussed above. The ratio of between the intensity of the aromatic CH stretching and the aliphatic CH2 stretching was then calculated for both the PET-DDT and PET-dDDT nanoparticles. For the former it was found to be 0.14 and for the latter it was found to be 0.13. We believe that these two values are within error one to each other indicating that the two particles have the same composition. We notice that these ratios do not indicated the stoichiometry on the ligand shell as they have not been corrected for the relative intensity of the two types of the peaks.

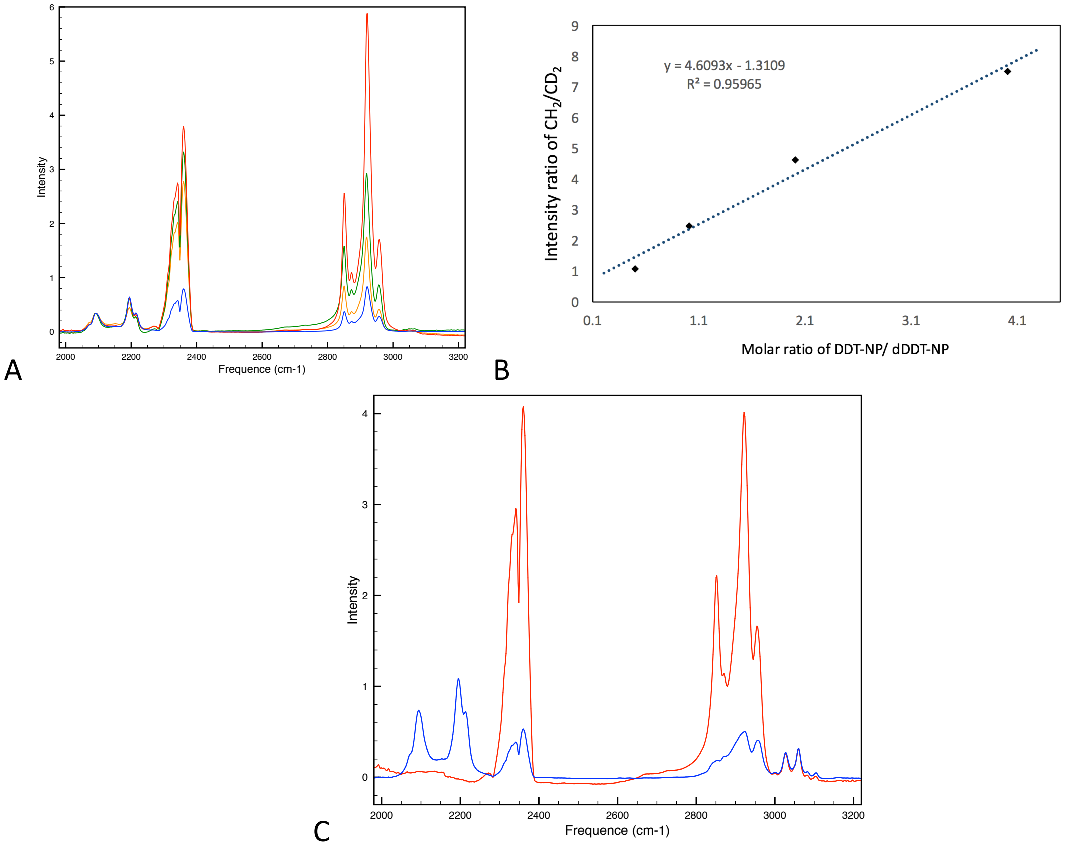

Supplementary Figure 2. FTIR analysis on the ligand ratio. A) FTIR spectra of DDT-NP and dDDT-NP mixtures at varying ratios from 0.3 to 0.8. B) Calibration curve of the CH_2/CD_2 peaks. C) FTIR spectra of the PET-DDT nanoparticle (red) and PET-dDDT nanoparticle (blue).

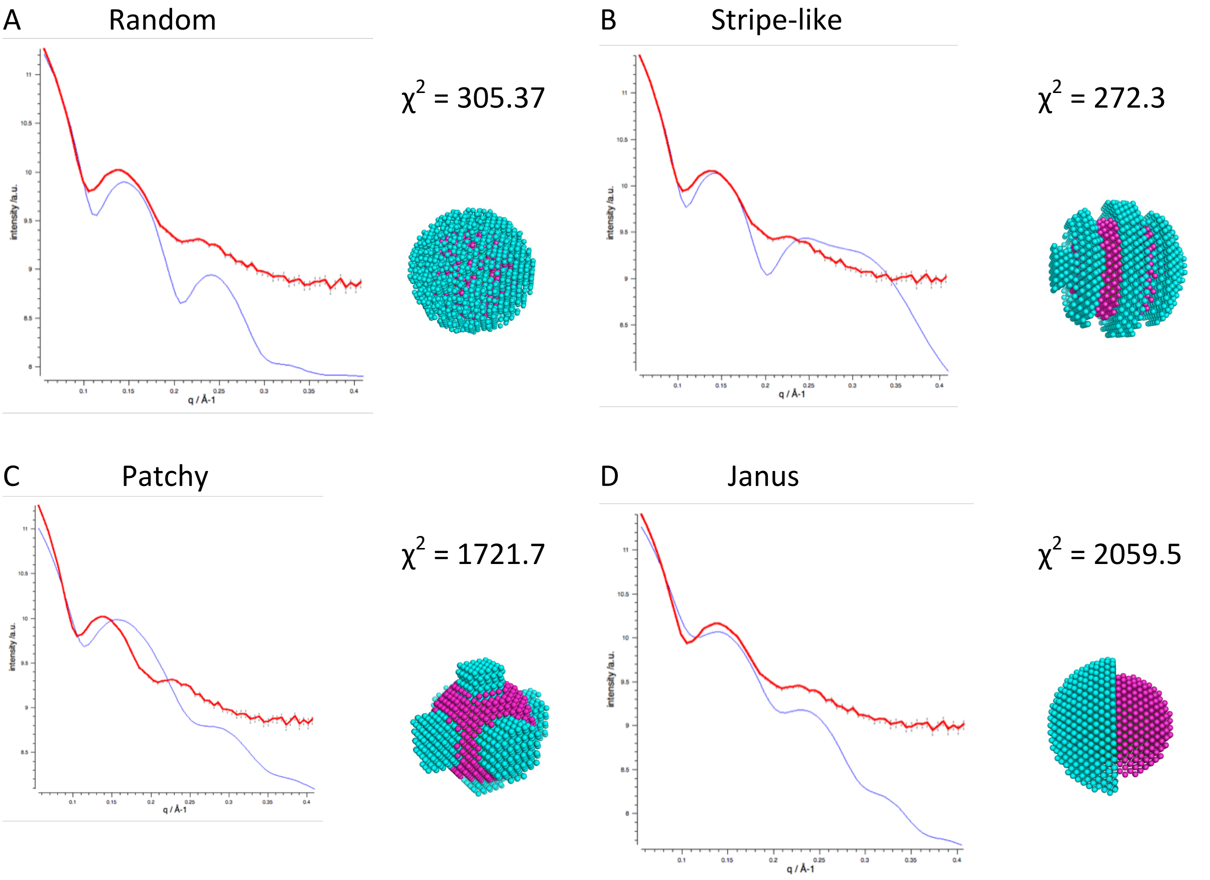

Supplementary Figure 3. Comparison between the experimental data and the theoretical scattering of known common ligand morphologies, i.e. A) Random; B) Stripe-like; C) Patchy; D) Janus.

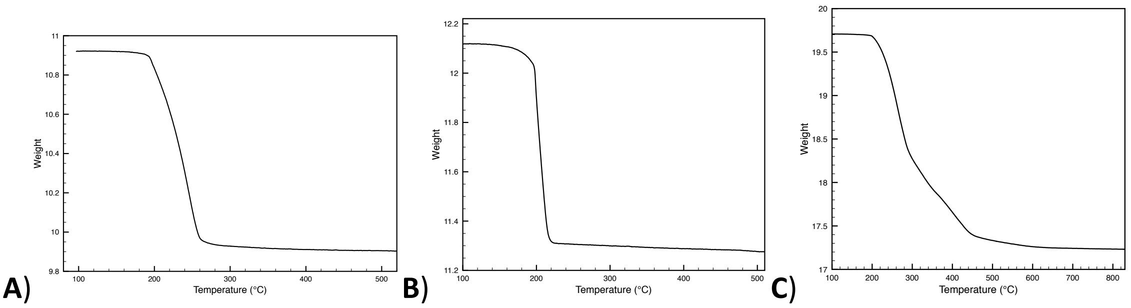

Supplementary Figure 4. A) TGA data of 5.5 nm PET-DDT nanoparticles; the ligand density is found to be 93.9% of the highest covering density on nanoparticle surface (i.e. each thiol occupies an area of 21.4 Å^2). B) TGA data of 4.4 nm PET-DDT nanoparticles; the ligand density is found to be 90.0% of the highest covering density. C) TGA data of 4 nm MUA-DDT nanoparticles; the ligand density is found to be 90.1% of the highest covering. The ligand ratio (1.3:1 MUA:DDT) could be estimated since the two ligands decompose at different temperature.

Supplementary Note 2. Description of MONSA fitting method

Scattering intensity from a dilute monodisperse solution is an average of scattering from all possible orientations of the particle, determined by $I(q)=\Sigma_{\Omega}(A^2(q))$, where $q=(4\pi \sin\theta)/\lambda$, is the scattering vector, while 2θ is scattering angle and λ is the wavelength of neutron or X-ray beam. In a SANS measurement, selective deuteration could be employed to highlight specific components. The scattering intensity thus becomes $I(q)=\Sigma_i^n(\Delta\rho_i V_i)^2 P_i\ (q)$, where $\Delta\rho_i=\rho_i-\rho_s$, is the contrast of component i against solvent and P_i (q) is the form factor of each component. The partial amplitudes from the volume occupied by the k-th phase in a DAM are rapidly evaluated from the bead positions using spherical harmonics.

Starting from a random configuration, simulated annealing (SA) is employed to search for a model composed by interconnected compact phases, which simultaneously fits multiple scattering curves from the constructs to minimize overall discrepancy:

$$\chi^2 = \sum_k \frac{1}{N_k - 1}\sum_{j=1}^{N_k}\left[\frac{I_{\text{exp}}^{(k)}(q_j) - c_k I_{\text{calc}}^{(k)}(q_j)}{\sigma^{(k)}(q_j)}\right]^2$$

Here the index k runs over the scattering curves, N_k are the numbers of experimental points, c_k are scaling factors and $I_{calc}(q_j)$ and $\sigma(q_j)$ are the intensities calculated from the subsets of the beads belonging to the appropriate phases and the experimental errors at the momentum transfer q_j, respectively.

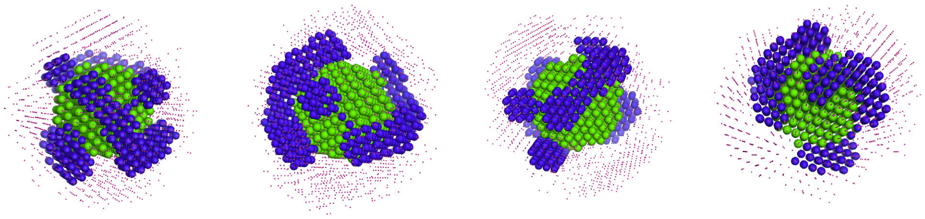

Supplementary Figure 5. Different projections of the model of the 4.4 nm PET: DDT=0.57: 1 nanoparticles.

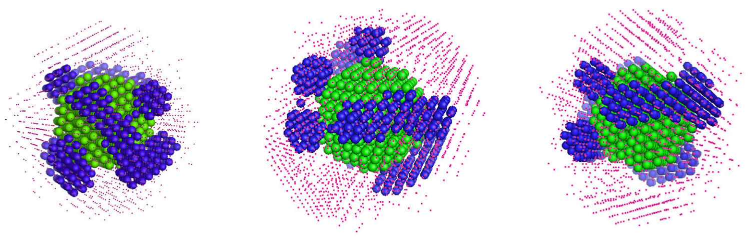

Supplementary Figure 6. Low-resolution models built from different starting random configurations.

Supplementary Note 3. Test of the Robustness of the Approach

To test the robustness of the proposed Monte-Carlo fitting method, different input parameters were used to fit the same data described above. Figure S7A shows the resulted model when the input volume fraction of PET-DDT was changed by 10% from NMR results, i.e. 22.9% for gold, 50.3% for DDT and 26.6% for PET. The low-resolution model, Figure S7A, built with these parameters still shows very similar features and the stripe-like elongated domain features remain substantially the same. When the volume fraction is further changed to more than 30%, the resulted model still shows elongated stripe-like domains, but the ligand shell structure becomes unphysical, with large bare-gold domains exposed to solvents. When no constraints are added to the volume fraction, i.e. volume fraction penalty is set to 0, but the gold core size is fixed, similar phase separation features are still observed (Figure S7B). Other input parameters such as looseness of beads are also changed and all the models are run several times starting from random configuration, yielding always similar results (Figure S7C).

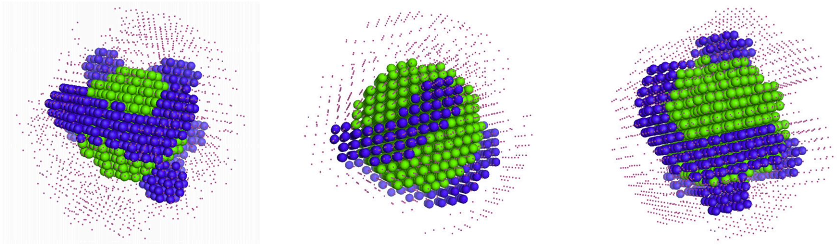

Supplementary Figure 7. 3D models built by varying the fitting parameters.

Supplementary Table 1. Scattering length values used in the SANS modeling.

Molecules	gold	CDCl3	DDT	PET	toluene
SLD(Å-2)	4.67E-06	3.16E-06	-3.68E-07	1.01E-06	9.41E-07
Molecules			dDDT	dPET	d-toluene
SLD(Å-2)			5.70E-06	4.52E-06	5.66E-06

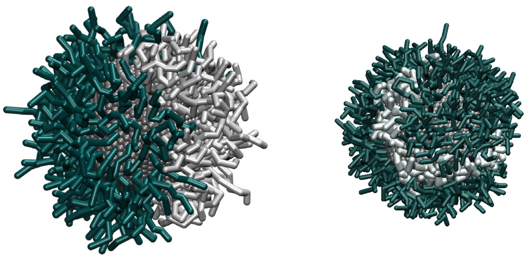

Supplementary Figure 8. DPD simulation of MUA-DDT (left) and MUA-BT (right) nanoparticles

Supplementary Note 4. Extracting averaged thickness of nanodomains

The averaged domain size was measured as shown below in Figure S6A. The domains composed of one type of the ligand, e.g. PET, is separated from the rest of the bead model. Then the thickness of each separate domain is then measured manually at different points. Histogram of the stripe thickness is then plotted and averaged thickness calculated.

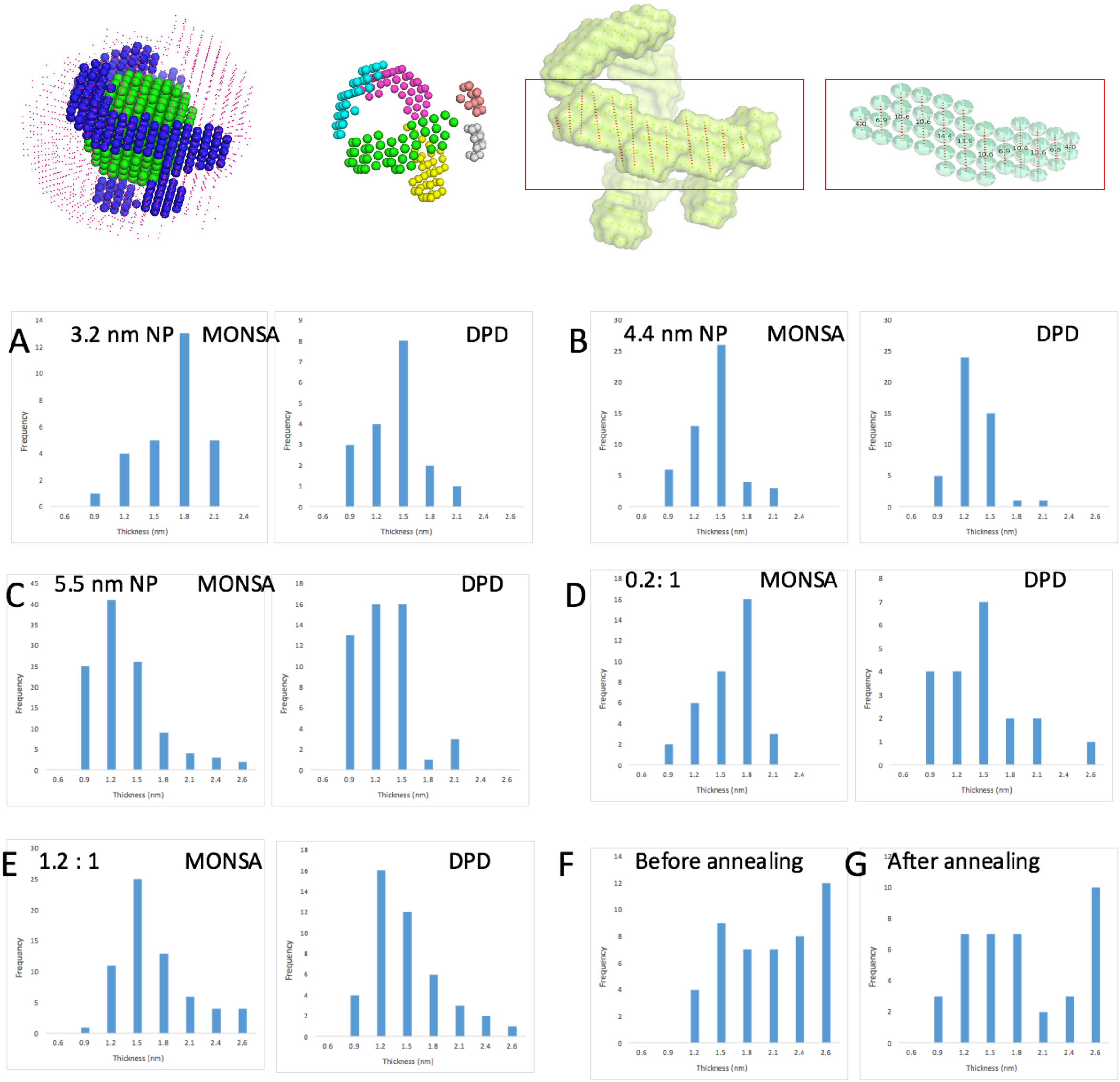

Supplementary Figure 9. Analysis of phase separation domain size from the 3D MONSA models and DPD simulation results. The analysis procedures and an example of measuring domain thicknesses were shown in the schemes. Distribution of stripe-like domain thickness for 3 nm (A), 4.4 nm (B) and 5.5 nm (C) PET-DDT nanoparticles and for 0.2: 1 (D) and 1.2: 1 (E) PET-DDT nanoparticles from MONSA (left) and simulation (right) models. And distribution of stripe-like domain thickness for PET-DDT nanoparticles before (F) and after annealing (G) from MONSA models.

Supplementary Note 5. Synthesis of Copper and Silver nanoparticles

Cu NPs were synthetized by following a two-step approach. First, TDPA ligand protected copper nanoparticles were synthesized following a reported procedure by L.I Hung et al.[L.-I Hung, C.-K. Tsung, W. Huang, P. Yang, Adv. Mater. 2010, 22, 1910.] In a typical synthesis, TOA (10 ml) was heated at 130C inside a three-neck flask for 30 min under a flow of N2 to remove water and dissolved O2. After cooling to room temperature, 1 mmol CuOAc and 0.5 mmol TDPA were added with vigorous stirring. The solution was flushed with N2, rapidly heated to 180C, maintained there for 30 min, rapidly heated to 270C, and then held there for an additional 30 min. The purplish-red solution was cooled to room temperature, mixed with ethanol and precipitated through centrifugation. The precipitate was dispersed in hexane. Then the purified nanoparticles were dissolved in toluene and 20 ul of dDDT ligand was added to the solution under stirring and reacted for 2 hours to allow for partial exchange. The nanoparticles were then precipitated with ethanol to remove extra ligands.

The silver nanoparticle synthesis followed the protocol reported by Stucky et. al. with small modifications. A 1: 1 mixture of toluene and chloroform was used as solvent to dissolve 55mg CF3COOAg to form a clear solution, to which 0.125 mmol of dPET and 0.125 mmol DDT were added. The solution was heated to 70 °C. Then, 217 mg borane t-butylamine complex was added into the solution under rapid stirring. 40ml methanol was added to quench the reaction after 1h. Nanoparticles were precipitated and then purified by repeated centrifugation and washing with methanol. Afterwards, intensity solvent fractionation was done using a mixture of ethanol and toluene solvent to minimize the size polydispersity of the nanoparticles. The final black precipitates were dried in vacuum overnight.

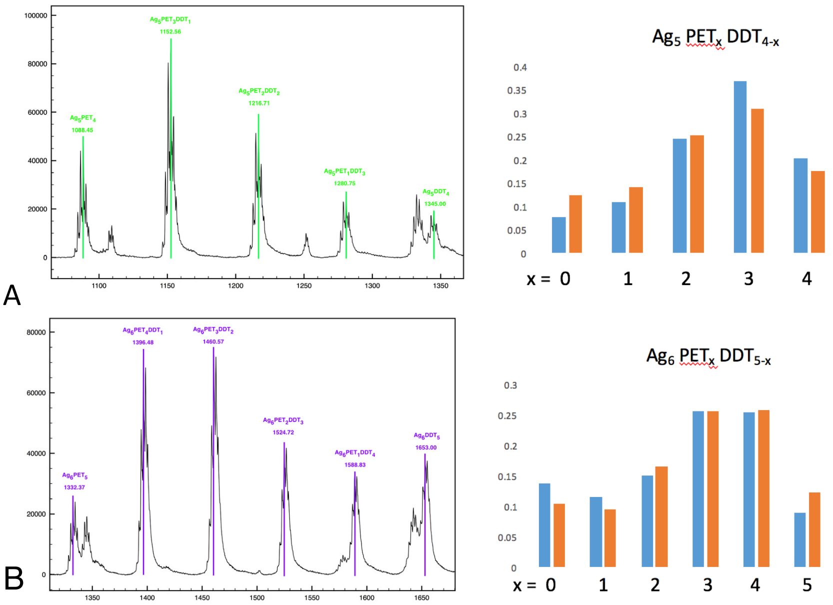

Supplementary Figure 10. MALDI-TOF MS data on PET-DDT silver nanoparticles (left) and the comparison with the expected MS pattern calculated from MONSA model (right). A) Ag_5 PET_x DDT_{4-x} data. B) Ag_6 PET_x DDT_{5-x} data.

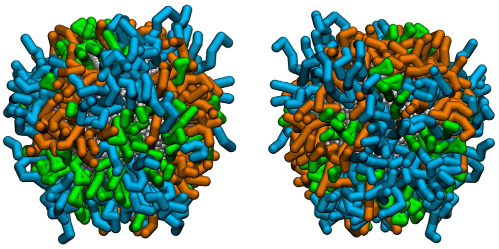

Supplementary Figure 11. Mesoscale morphology resulting from DPD simulations of the PET-OT-DDT nanoparticles. Green sticks represent PET ligands, orange sticks represent OT ligands and light blue is used for DDT chains. Solvent is not shown for clarity.

Supplementary Note 6. Computational methods

Dissipative Particle Dynamics (DPD)

DPD[1] is a particle-based mesoscopic simulation technique extensively used to model block copolymers[2,3], mesophases[4], surfactants[5-7] and polymer phase separations[8], as well as the assembly of patchy and striped patterns on monolayer protected nanoparticle[9-11].

The DPD particles (or beads), each representing a group of small molecules or part of a molecule, interact by conservative, dissipative, and random forces, which are pairwise additive. The net force acting on a bead *i* can be expressed as $\boldsymbol{F}_i = \sum_{j\neq i} (\boldsymbol{F}_{ij}^C+\boldsymbol{F}_{ij}^D+\boldsymbol{F}_{ij}^R)$ and is calculated by summation over all other particles within a certain cutoff radius, r_c, which gives the extent of the interaction range. Let r_c, m, and k_BT be the unit distance, the particle mass, and the thermal energy, respectively.

The conservative force represents the excluded volume interactions between particles *i* and *j* in the dimensionless form $\boldsymbol{F}_{ij}^C = a_{ij}\,(1 - r_{ij}\,)\,\hat{\boldsymbol{r}}_{ij}$, where $\boldsymbol{r}_{ij} = \boldsymbol{r}_i - \boldsymbol{r}_j$, $r_{ij} = |\boldsymbol{r}_{ij}|$, $\hat{\boldsymbol{r}}_{ij} = \boldsymbol{r}_{ij}/r_{ij}$, a_{ij} is the maximum repulsion between particles *i* and *j*. The dissipative, $\boldsymbol{F}_{ij}^D = -\,\gamma\,\omega(r_{ij})^2\,(\hat{\boldsymbol{r}}_{ij}\cdot\boldsymbol{v}_{ij})\,\hat{\boldsymbol{r}}_{ij}$, and random forces, $\boldsymbol{F}_{ij}^R = \sigma\,\omega(r_{ij})\,\hat{\boldsymbol{r}}_{ij}\,\zeta/(\delta t)^{-1/2}$, act as heat sink and source, respectively, and the combined effect of the two forces performs as a thermostat, where γ is a friction coefficient related to the thermal noise amplitude σ via the fluctuation–dissipation theorem, $\sigma^2 = 2\gamma k_BT$, $\omega(r)$ is a weight function, ζ is a normally distributed random variable with zero mean and unit variance that is uncorrelated for different particle pairs, δt is the time step of an integration scheme, and $\boldsymbol{v}_{ij} = \boldsymbol{v}_i - \boldsymbol{v}_j$ is the relative velocity of the ith and the jth particles. The equations of particle motion, $d\boldsymbol{r}_i/dt = \boldsymbol{v}_i$ and $d\boldsymbol{v}_i/dt = \boldsymbol{F}_i$, are solved using as integration scheme the velocity-Verlet algorithm implemented in LAMMPS[12-14].

Finally, when modelling chains two additional forces are acting between bonded beads: a harmonic spring connecting two adjacent particles *i* and *j* $\boldsymbol{F}_{ij}^B = k_b(r_{ij} - r_0)\,\hat{\boldsymbol{r}}_{ij}$, where k_b is a spring constant and r_0 the equilibrium distance between the particles, and $\boldsymbol{F}_{ijz}^A = 1/2\ k_\vartheta \sin(\vartheta_0\text{-}\vartheta_0)$, where k_ϑ is a spring constant and ϑ_0 the equilibrium angle between adjacent beads triples *ijz* in a row.

Models and computational details

The initial structure of the nanoparticle core was constructed by arranging gold coarse-grained units (or DPD beads) on an *fcc* lattice into an icosahedron of the desired diameter using OPENMD software (v. 2.3)[15]. Each ligand was represented by a flexible chain model of

beads connected by harmonic springs. The mesoscale topology was assessed by matching the atomistic (calculated by molecular dynamics (MD) simulations) and mesoscale pair correlation functions for each ligand chain[16]. This resulted in the following mesoscale chains for MUA, DDT, PET, BT, OT: $S_1CM_5CA_1$, S_1CD_6, $S_1CP_1B_3$, S_1CB_2, S_1CO_4, respectively. Bead of type S typically represents the sulphur head group, beads CX (where X varies according to the ligand) denote the hydrocarbon units, CA unit mimics the carboxylic moiety. Benzene groups were modelled as a three-beads B rings. The subscripts indicate the bead number for each bead type.

Each ligand was placed close to the NP surface and oriented outward with the head-tail vector along the radial direction, ensuring that the corresponding position on the surface did not have clash with any previously positioned ligand using Packmol package[17]. A random configuration was imposed to arrange the chains on the gold surface. Then, the modified NP was solvated again by Packmol. Each system was tested on three independently generated starting configurations. To ascertain that the morphologies obtained correspond to a thermodynamic equilibrium, simulations were performed starting also from a phase-separated configuration of the ligands.

The intra- and intermolecular interactions between DPD particles are expressed by the conservative parameter a_{ij}, which inherits the chemical information of the system, and were estimated from atomistic MD simulations rescaling the MD interaction energies onto the corresponding mesoscale a_{ij} parameter values[11,16,18-21] based on the modified PCFF force field (INTERFACE FF), optimized by Heinz and co-workers[22] for hybrid organic and metal interfaces, including gold. According to this computational procedure, two bead-bead interaction parameters have to be chosen. The gold-gold interaction parameter was set to $a_{Au\text{-}Au}$ = 49.6, based on our previous calculations[11,21] while we assigned toluene-toluene and tetrahydrofuran-tetrahydrofuran interaction equal to $a_{TOL\text{-}TOL}$= 13.7 and $a_{THF\text{-}THF}$= 35.1, respectively, based on the direct relationship with their isothermal compressibility at room temperature[23]. Therefore, we employed the following values for **MUA-DDT** particles: $a_{S\text{-}S}$ = 48.1, $a_{S\text{-}CM}$ = 72.6, $a_{CM\text{-}CM}$ = 44.1, $a_{S\text{-}CA}$ = 75.1, $a_{CA\text{-}CM}$ = 85.2, $a_{CA\text{-}CA}$ = 40.6, $a_{S\text{-}CD}$ = 72.9, $a_{CD\text{-}CM}$ = 43.8, $a_{CD\text{-}CA}$ = 85.8, $a_{CD\text{-}CD}$ = 43.6, $a_{S\text{-}THF}$ = 68.7, $a_{THF\text{-}CM}$ = 58.0, $a_{THF\text{-}CA}$ = 49.7, $a_{THF\text{-}CD}$ = 58.2, $a_{Au\text{-}S}$ = 1.9, $a_{Au\text{-}CM}$ = 58.6, $a_{Au\text{-}CA}$ = 65.5, $a_{Au\text{-}CD}$ = 58.4, $a_{Au\text{-}THF}$ = 60.7. The interaction parameters for **MUA-BT** nanoparticles were: $a_{S\text{-}S}$ = 48.1, $a_{S\text{-}CM}$ = 72.6, $a_{CM\text{-}CM}$ = 44.1, $a_{S\text{-}CA}$ = 75.1, $a_{CA\text{-}CM}$ = 85.2, $a_{CA\text{-}CA}$ = 40.6, $a_{S\text{-}CB}$ = 70.6, $a_{CB\text{-}CM}$ = 58.2, $a_{CB\text{-}CA}$ = 75.3, $a_{CB\text{-}CB}$ = 46.9, $a_{S\text{-}THF}$ = 68.7, $a_{THF\text{-}CM}$ = 58.0, $a_{THF\text{-}CA}$ = 49.7, $a_{THF\text{-}CB}$ = 56.9, $a_{Au\text{-}S}$ = 1.9, $a_{Au\text{-}CM}$ = 58.6, $a_{Au\text{-}CA}$ = 65.5, $a_{Au\text{-}CB}$ = 59.9, $a_{Au\text{-}THF}$ = 60.7.
In the case of **PET-DDT** particles: $a_{S\text{-}S}$ = 48.2, $a_{S\text{-}CP}$ = 70.1, $a_{CP\text{-}CP}$ = 52.0, $a_{S\text{-}B}$ = 72.2, $a_{B\text{-}CP}$ = 57.3, $a_{B\text{-}B}$= 56.0, $a_{S\text{-}CD}$ = 72.6, $a_{CD\text{-}CP}$ = 57.9, $a_{CD\text{-}B}$ = 57.1, $a_{CD\text{-}CD}$ = 52.1, $a_{S\text{-}TOL}$ = 73.5, $a_{TOL\text{-}CP}$ = 52.9, $a_{TOL\text{-}B}$ = 51.7, $a_{TOL\text{-}CD}$ = 57.8, $a_{Au\text{-}S}$ = 1.8, $a_{Au\text{-}CP}$ = 57.6, $a_{Au\text{-}B}$ = 56.7, $a_{Au\text{-}CD}$ = 58.3 $a_{Au\text{-}TOL}$ = 58.2.The last set of parameters for **PET-OT-DDT** particles is composed by the following values: $a_{S\text{-}S}$ = 48.2, $a_{S\text{-}CP}$ = 70.1, $a_{CP\text{-}CP}$ = 55.1, $a_{S\text{-}B}$ = 72.2, $a_{B\text{-}CP}$ = 55.5, $a_{B\text{-}B}$= 61.0, $a_{S\text{-}CD}$ = 72.6, $a_{CD\text{-}CP}$ = 61.3, $a_{CD\text{-}B}$ = 61.6, $a_{CD\text{-}CD}$ = 52.6, $a_{CO\text{-}S}$ = 71.5, $a_{CO\text{-}CP}$ = 60.5, $a_{CO\text{-}B}$ = 60.1, $a_{CO\text{-}CD}$ = 57.2, $a_{CO\text{-}COB}$= 53.2, $a_{S\text{-}TOL}$ = 73.5, $a_{TOL\text{-}CP}$ = 52.9, $a_{TOL\text{-}B}$ = 51.7, $a_{TOL\text{-}CD}$ = 57.8, $a_{TOL\text{-}CO}$ = 57.0, $a_{Au\text{-}S}$ = 1.8, $a_{Au\text{-}CP}$ = 57.6, $a_{Au\text{-}B}$ = 56.7, $a_{Au\text{-}CD}$ = 58.3, $a_{Au\text{-}CO}$ = 57.9, $a_{Au\text{-}TOL}$ = 58.2.
Optimized adimensional values for bond and angle parameters were employed for the thiols: k_b(CX-CX) = 10, r_0(CX-CX) = 0.55, k_ϑ(CX-CX-CX) = 10, ϑ_0(CX-CX-CX) = 110, k_b(B-B) = 40, r_0(B-B) = 0.49, k_ϑ(B-B-B) = 40, ϑ_0(B-B-B) = 60, k_b(B-CP) = 40, r_0(B-CP) = 0.39, ϑ_0(B-B-CP) = 150

where k_b and k_ϑ are the bond and angle spring constants while r_0 and ϑ_0 are the equilibrium distance and angle between connected beads, respectively.

Each configuration was first relaxed for 1×10^4 steps and a time step of Δt = 0.01τ. Then, at least additional 6×10^6 time steps (Δt = 0.02τ) were performed for productive runs. System equilibration was assessed monitoring temperature, pressure, density, and potential energy behaviour as well as composition of nearest neighbours of head groups.

The force cut-off radius r_c, the particle mass m_i, and k_BT (where are k_B is the Boltzmann factor and T is the temperature) were taken as units of length, mass and energy. All mesoscale production runs, analysis and imaging were performed using LAMMPS running on GPUs and VMD[24].

Supplementary Note 7. Synthesis of deuterated ligands

Synthesis of 11-mercaptoundecanoic-d_{18} acid

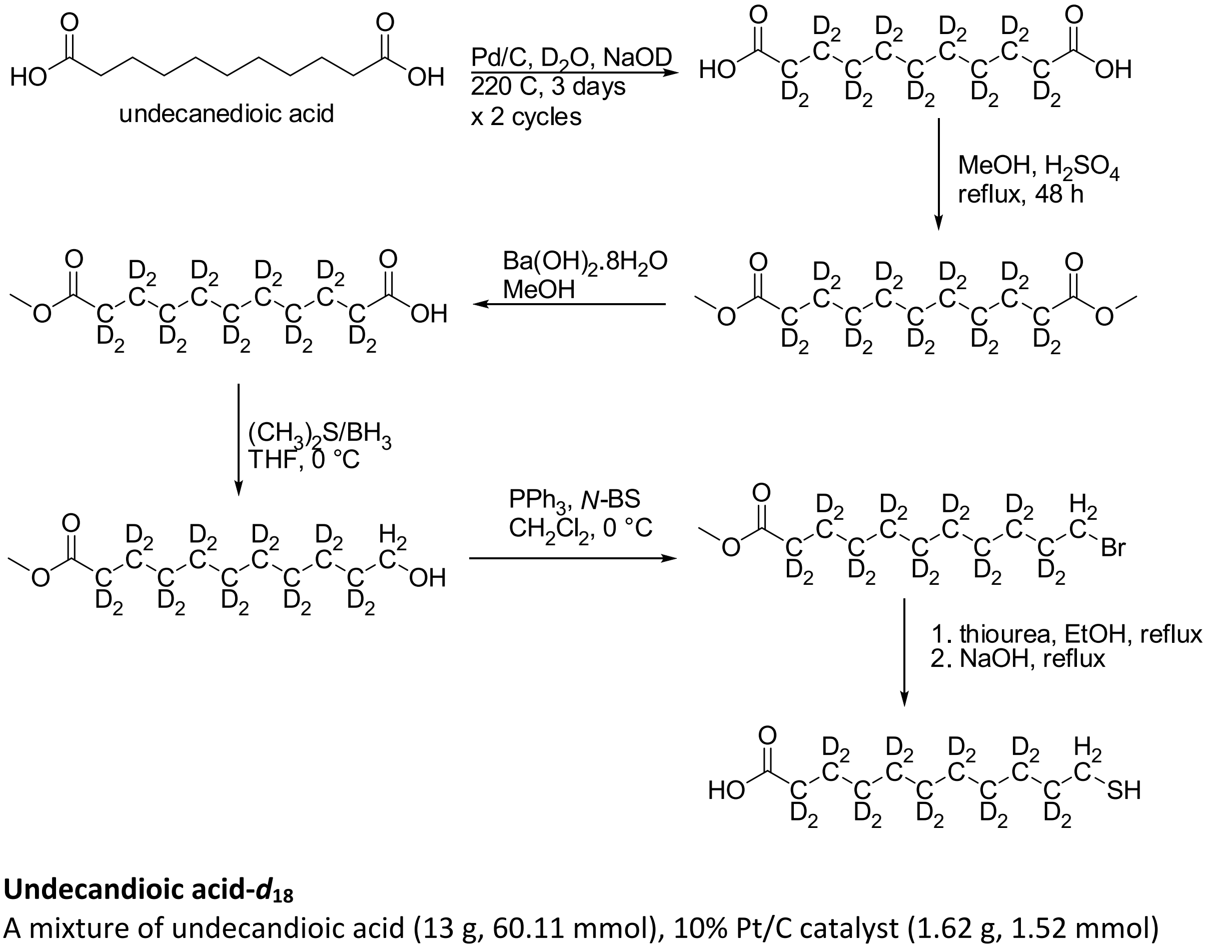

Undecandioic acid-d_{18}

A mixture of undecandioic acid (13 g, 60.11 mmol), 10% Pt/C catalyst (1.62 g, 1.52 mmol) and 40% NaOD solution (4.92 g, 120.22 mmol or 12.32 mL of 40% NaOD in D_2O) in D_2O (120 mL) was stirred under hydrothermal conditions for 72 hours at 220 °C in a Mini Benchtop 4560 Parr reactor (600 mL vessel capacity, 206 bar max. pressure, 350 °C max. temperature). After cooling, the reaction mixture was diluted with dichloromethane (200 mL) and the mixture filtered through Celite to remove the catalyst. The filtered catalyst was washed with water (3×100 mL) and the filtrate acidified to pH 2 with dilute HCl. The product

was extracted with diethyl ether (100 mL×3) and the combined organic phases were dried over Na_2SO_4, filtered and concentrated in *vacuo* to give a white solid (12.5 g). The product was then run through a second cycle in the Parr reactor with fresh Pt/C catalyst, NaOD and D_2O under the same conditions as above. Following extraction and drying, the final product was obtained as a white solid (10.6 g, 45.07 mmol, 75% yield, 97%D). ESI-MS (-ve mode) m/z: predicted $[M-H]^-$ $C_{11}H_2D_{18}O_4$: 233.3, found: 233.3; overall 97% D with isotopic distribution: d_{16} 9.3%, d_{17} 32.3%, d_{18} 58.3%. (Figure 12).

Dimethyl undecanedioate-d_{18}

Following a modified method of Darwish et al.[1], undecandioic acid-d_{18} (11.0g, 46.9 mmol) was dissolved in anhydrous methanol (150 mL), to which concentrated sulfuric acid (3 mL, excess) was added and the solution heated to reflux for 48 h. The majority of the methanol was removed under reduced pressure and poured onto an ice/water slurry. This was extracted with diethyl ether (2 × 200 mL), with the combined organics washed with $NaHCO_3$ solution, brine, dried over Na_2SO_4 and evaporated under reduced pressure to give a white solid (10.6 g, 86%).

^{1}H NMR (400 MHz, $CDCl_3$): 3.66 (6H, 2 × CH_3), 2.25 (residual 2 x CH_2), 1.56 (residual 2 x CH_2), 1.23 (residual 5 x CH_2). ^{2}H NMR (60 MHz, $CDCl_3$): 2.26 (4D, 2 x CD_2), 1.56 (4D, 2 x CD_2), 1.23 (10D, 5 x CD_2). ^{13}C NMR {^{1}H, ^{2}H decoupled} (100 MHz, $CDCl_3$): 174.5 (2 x C=O), 51.5 (CH_3), 33.4 (CD_2), 27.9 (3 x CD_2), 23.9 (CD_2) (Figures 13-15)

11-methoxy-11-oxoundecanoic acid-d_{18}

Following a modified method of Darwish et al.[1], dimethyl undecanedioate-d_{18} (10.6 g, 40.4 mmol) was dissolved in anhydrous methanol (20 mL). Barium hydroxide hexahydrate (6.5 g, 20.6 mmol, 0.51 equiv.) was partially dissolved in anhydrous methanol (20 mL) and added to the solution of the diester. The suspension was stirred at room temperature for 24 h. The solid was collected via vacuum filtration and washed with cold methanol (2 x 50 mL). The solid was suspended in water (150 mL) with stirring, to which HCl (1 M) was added until the solid dissolved. The solution was extracted with diethyl ether (2 x 150 mL). The combined organics were washed with brine, dried over Na_2SO_4 and evaporated under reduced pressure to give a clear, colourless oil (7.2 g, 72% crude yield). ^{1}H NMR analysis (Figure 16) showed the product contained ~16% of the undesired diacid due to the disproportion in the integration values of the residual proton signals of the methylene units next to the acid and the ester groups. It was decided to continue on to the next step without purification.

^{1}H NMR (400 MHz, $CDCl_3$): 3.66 (3H, CH_3), 2.30 (0.088H, residual CH_2), 2.26 (0.058H, residual CH_2), 1.57 (residual 2 x CH_2), 1.27 (residual 5 x CH_2). (residual 2 x CH_2). ^{2}H NMR (60 MHz, $CDCl_3$): 2.26 (4D, 2 x CD_2), 1.56, (4D, 2 x CD_2), 1.23 (4D, 5 x CD_2). ^{13}C NMR {^{1}H, ^{2}H decoupled} (100 MHz, $CDCl_3$): 180.1 (1 x C=O, acid), 174.6 (1 x C=O, ester), 51.6 (CH_3), 33.3 (CD_2), 27.9 (2 x CD_2), 24.0 (CD_2), 23.7 (CD_2). (Figures 16-18)

Methyl 11-hydroxyundecanoate-d_{18}

Following a modified method of Darwish et al.[1], crude 11-methoxy-11-oxoundecanoic acid-d_{18} (6.8 g, 27.4 mmol), was dissolved in anhydrous THF (50 mL) and cooled to 0 °C, to which borane-methyl sulfide complex (10 M, 3.8 mL, 38 mmol), was added drop-wise under an inert atmosphere. The solution was maintained at 0 °C for 2 h and allowed to come to room temperature overnight. Methanol (5 mL) was added dropwise and stirred for a further 2 h. The solution was sparged with $N_{2(g)}$ and the solvent removed under reduced pressure.

Water and diethyl ether were added, with the aqueous layer extracted 2x with diethyl ether. The combined organics were sequentially washed with $NaHCO_3$ solution, brine and dried over Na_2SO_4 and evaporated to give a clear oil. The oil was purified on silica by automated chromatography using petroleum ether/ethyl acetate mobile phase, to give a clear, colourless oil (3.7 g, 58%).

^{1}H NMR (400 MHz, $CDCl_3$): 3.65 (3H, CH_3), 3.60 (2H, CH_2), 2.26 (residual 2 x CH_2), 1.49 (residual 2 x CH_2), 1.23 (residual 5 x CH_2). ^{2}H NMR (60 MHz, $CDCl_3$): 2.26 (4D, 2 x CD_2), 1.56 (4D, 2 x CD_2), 1.23 (10D, 5 x CD_2). ^{13}C NMR {^{1}H, ^{2}H decoupled} (100 MHz, $CDCl_3$): 174.6 (C=O), 63.0 (CH_2-OH), 51.5 (CH_3), 33.4 (CD_2), 31.8 (CD_2), 28.3 (CD_2), 28.2 (CD_2), 28.1 (CD_2), 28.0 (CD_2), 27.0 (CD_2), 24.6 (CD_2), 24.0 (CD_2). (Figures 19-21)

Methyl 11-bromoundecanoate-*d_{18}*

Methyl 11-hydroxyundecanoate-d_{18} (3.7 g, 15.8 mmol) was dissolved in anhydrous dichloromethane (80 mL), to which triphenylphosphine (6.2 g, 23.7 mmol, 1.5 equiv.) was added. The mixture was cooled in an ice bath and stirred for 45 min. *N*-Bromosuccinimide (4.2 g, 23.7 mmol, 1.5 equiv.) was added portion-wise, and the solution stirred at 0 °C for 2 h and then allowed to warm to room temperature. The dark orange solution was evaporated to remove the CH_2Cl_2, and petroleum ether added. The white precipitate (triphenylphosphine oxide) was removed via filtration, and the filtrate concentrated and purified on silica by automated chromatography using petroleum ether/ethyl acetate mobile phase, to give a clear, pale yellow oil (3.0 g, 64%).

^{1}H NMR (400 MHz, $CDCl_3$): 3.66 (3H, CH_3), 3.38 (2H, CH_2), 2.26 (residual 1 x CH_2), 1.81 (residual 1 x CH_2), 1.56 (residual 1 x CH_2), 1.35 (residual 1 x CH_2), 1.22 (residual 5 x CH_2). ^{2}H NMR (60 MHz, $CDCl_3$): 2.26 (2D, 1 x CD_2), 1.80 (2D, 1 x CD_2), 1.56 (2D, 1 x CD_2), 1.36 (2D, 1 x CD_2), 1.22 (10D, 5 x CD_2). ^{13}C NMR {^{1}H, ^{2}H decoupled} (100 MHz, $CDCl_3$): 174.5 (C=O), 51.5 (CH_3), 34.0(CH_2-Br), 33.4 (CD_2), 32.0 (CD_2), 28.1 (x2 CD_2), 28.0 (CD_2), 27.9 (CD_2), 27.1 (CD_2), 24.0 (CD_2). (Figures 22-24)

11-mercaptoundecanoic acid-*d_{18}*

Followed a modified procedure,[2] methyl 11-bromoundecanoate-d_{18} (3.0 g, 10.1 mmol), and thiourea (0.81 g, 10.6 mmol, 1.05 equiv.) were refluxed in absolute ethanol (15 mL) for 2 h. Upon cooling to room temperature, a solution of NaOH (3.2 g, 40.4 mmol, 4 equiv., 10 mL) was added dropwise, and the solution stirred overnight at room temperature. The cloudy solution was refluxed for a further 3 h, and upon cooling the precipitate was removed via filtration and dried under vacuum. The solid was recrystallised from hexane to give a cream coloured solid (1.84 g, 77%).

^{1}H NMR (400 MHz, $CDCl_3$): 2.50 (d, 2H, C$\underline{H_2}$-SH), 2.31 (residual 1 x CH_2), 1.58 (residual 2 x CH_2), 1.32 (t, 1H, CH_2-S$\underline{H}$), 1.22 (residual 6 x CH_2). ^{2}H NMR (60 MHz, $CDCl_3$): 2.31 (2D, 1 x CD_2), 1.57 (4D, 2 x CD_2), 1.22 (12D, 6 x CD_2). ^{13}C NMR {^{1}H, ^{2}H decoupled} (100 MHz, $CDCl_3$): 180.2 (C=O), 33.3 (CD_2), 33.1 (CD_2), 28.2 (CD_2), 28.1 (CD_2), 28.0 (CD_2), 27.8 (CD_2), 27.2 (CD_2), 24.6 (CH_2), 23.7 (CD_2). (Figures 25-27)

ESI-MS m/z $C_{11}H_4D_{18}O_2S$ predicted: 235.3 $[M-H]^-$, found 235.3. The overall level of deuteration of the non-exchangeable protons in 11-mercaptoundecanoic acid-d_{20} (20

positions) is 87.7% D with isotopic distribution d_{15} 1.7%, d_{16} 7.8%, d_{17} 30.6%, d_{18} 55.1%. (Figures 28-29).

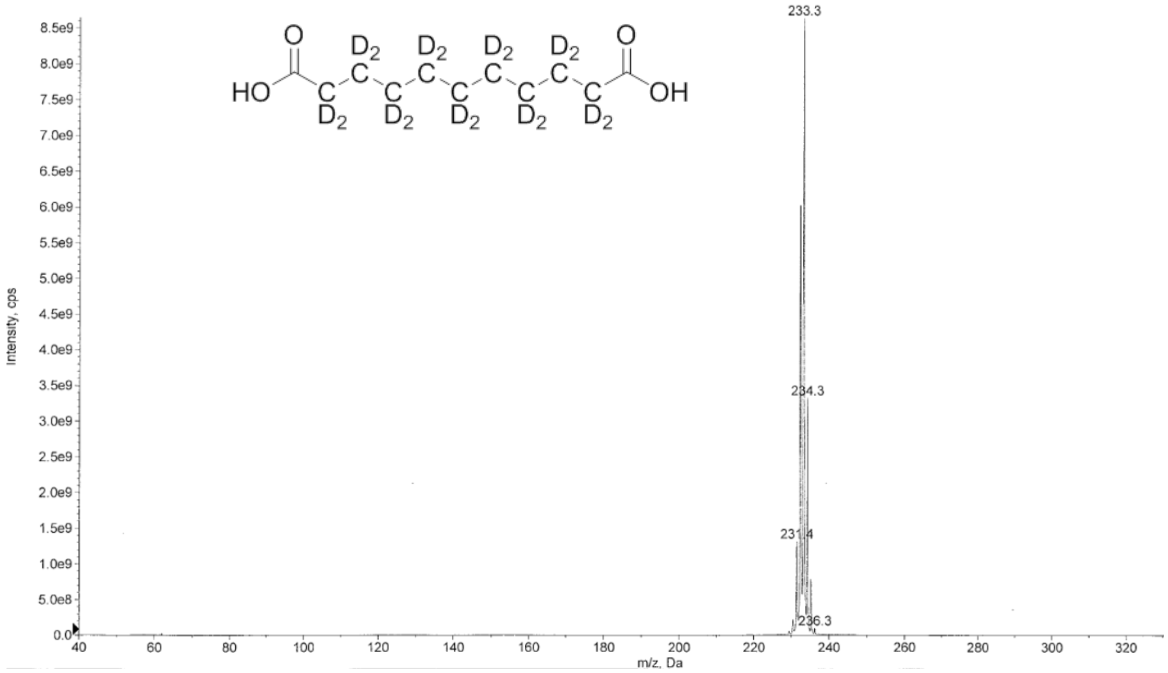

Supplementary Figure 12. ESI-MS (-ve mode), enhanced resolution (ER) spectrum of Undecandioic acid-d_{18}

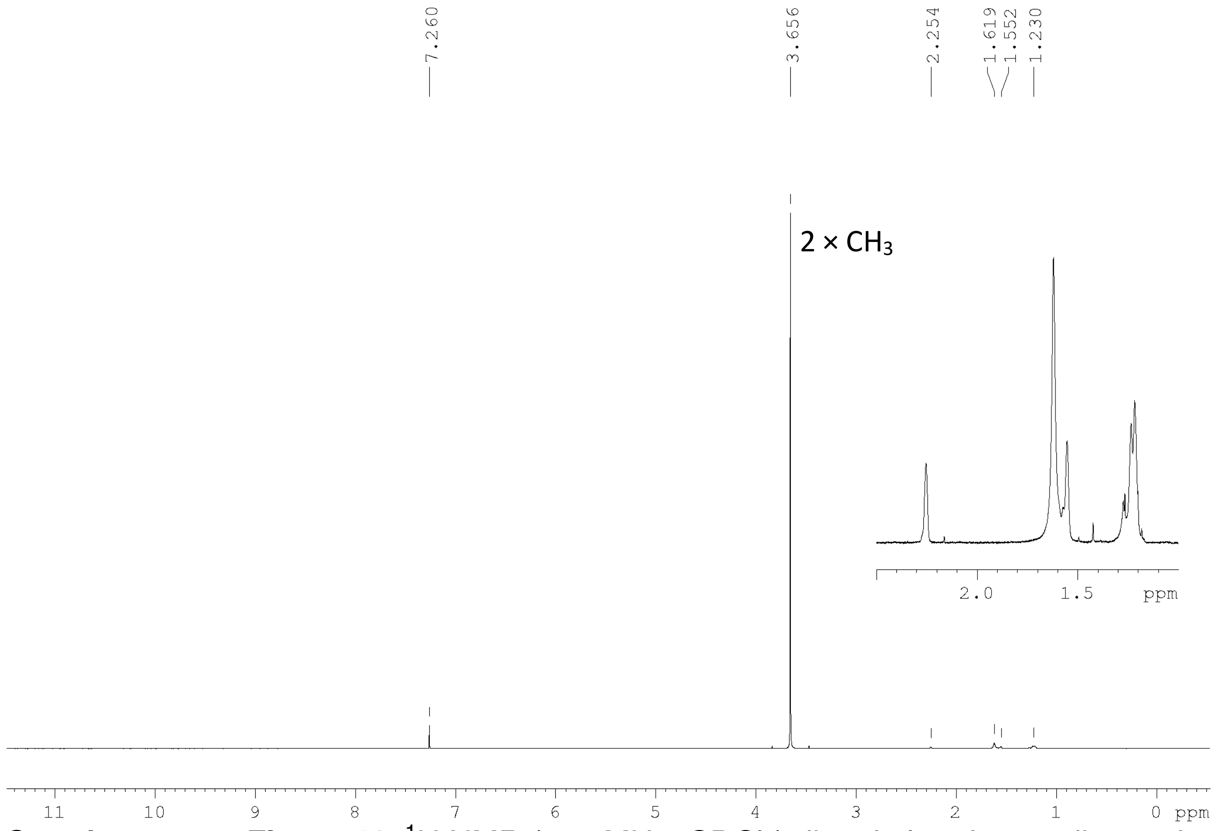

Supplementary Figure 13. ^{1}H NMR (400 MHz, $CDCl_3$) dimethyl undecanedioate-d_{18}

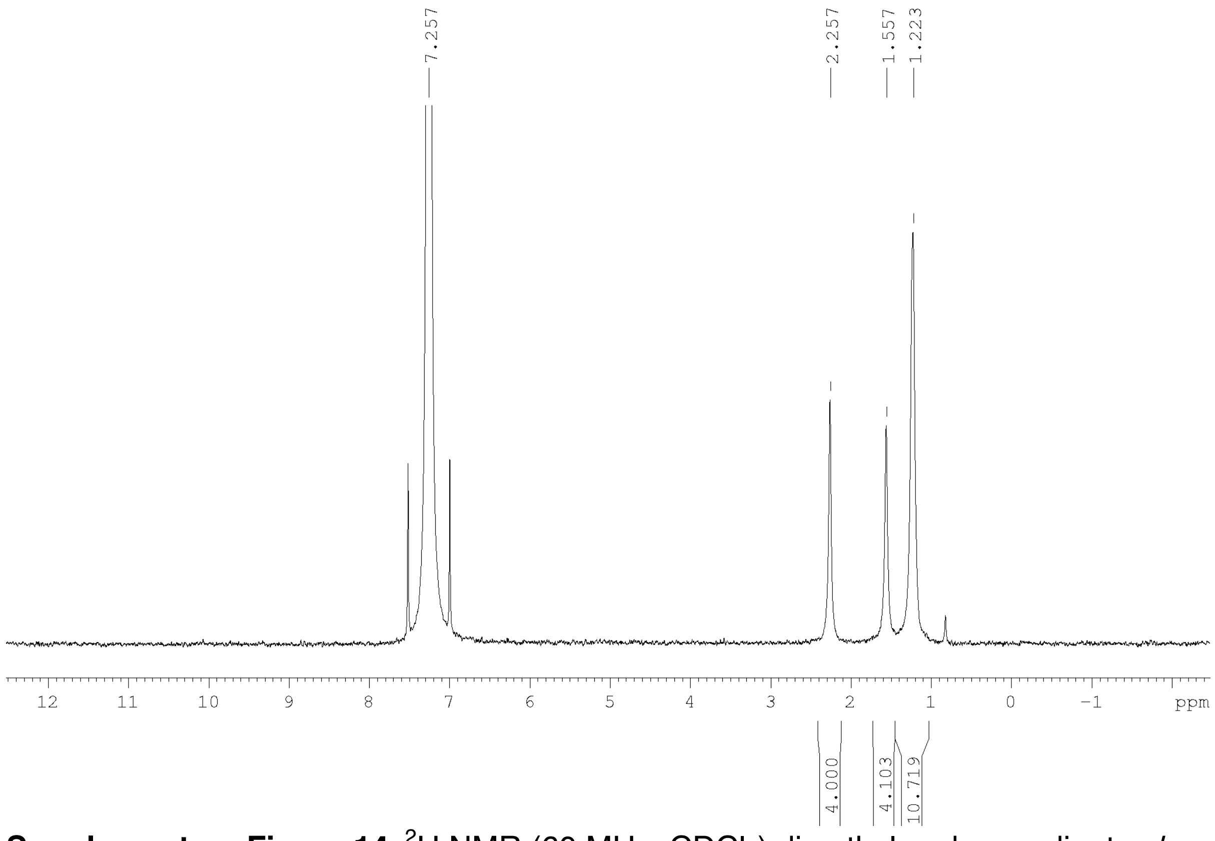

Supplementary Figure 14. ^{2}H NMR (60 MHz, $CDCl_3$) dimethyl undecanedioate-d_{18}

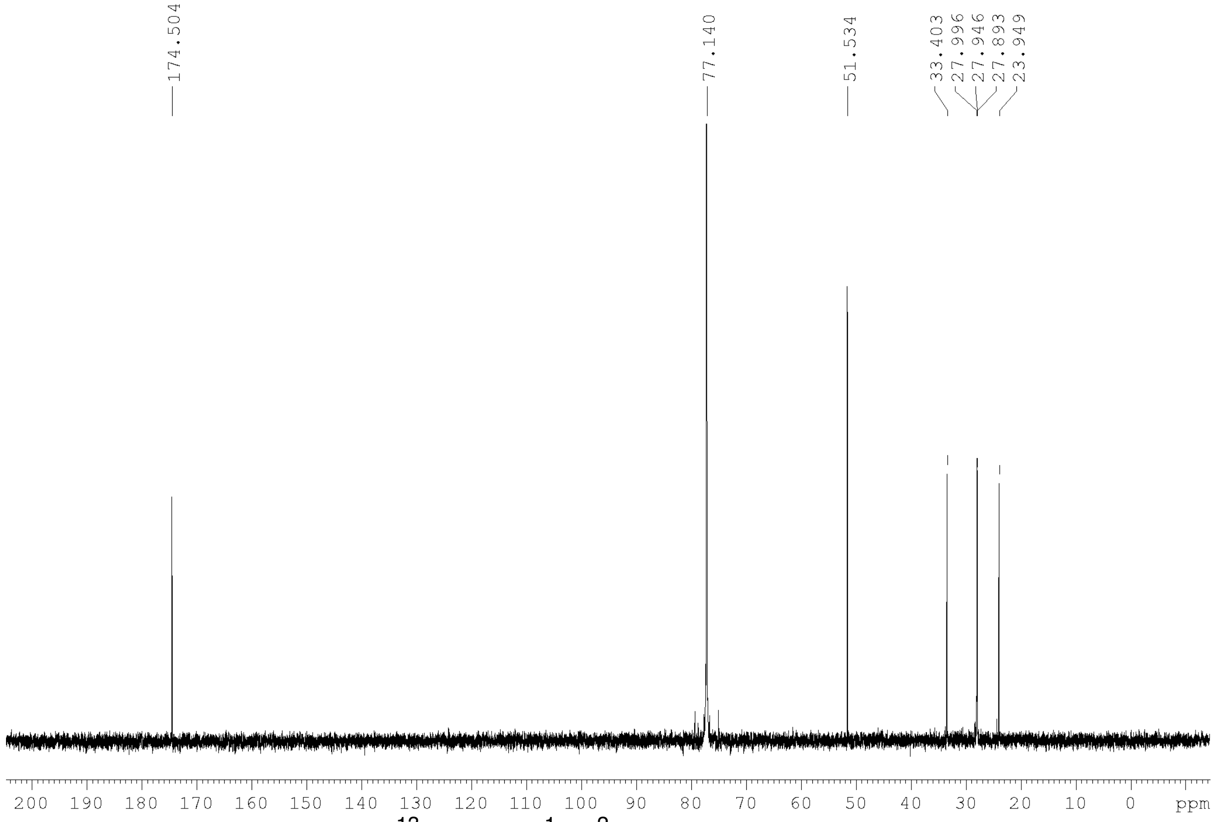

Supplementary Figure 15. ^{13}C NMR {^{1}H, ^{2}H decoupled} (100 MHz, $CDCl_3$) dimethyl undecanedioate-d_{18}

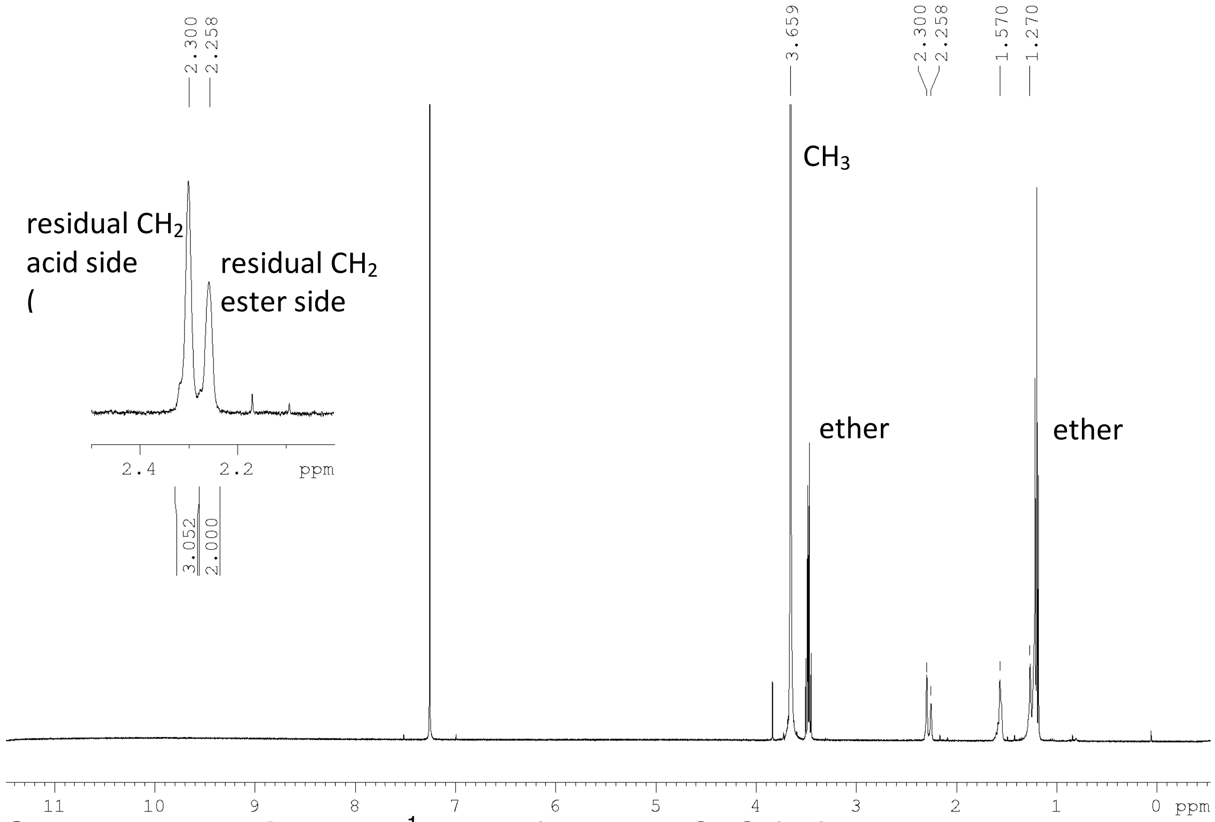

Supplementary Figure 16. ^{1}H NMR (400 MHz, $CDCl_3$) of 11-methoxy-11-oxoundecanoic acid-d_{18}. Inset is the relative integration ratio of the residual protons signals of the methylene units next to the acid and ester groups.

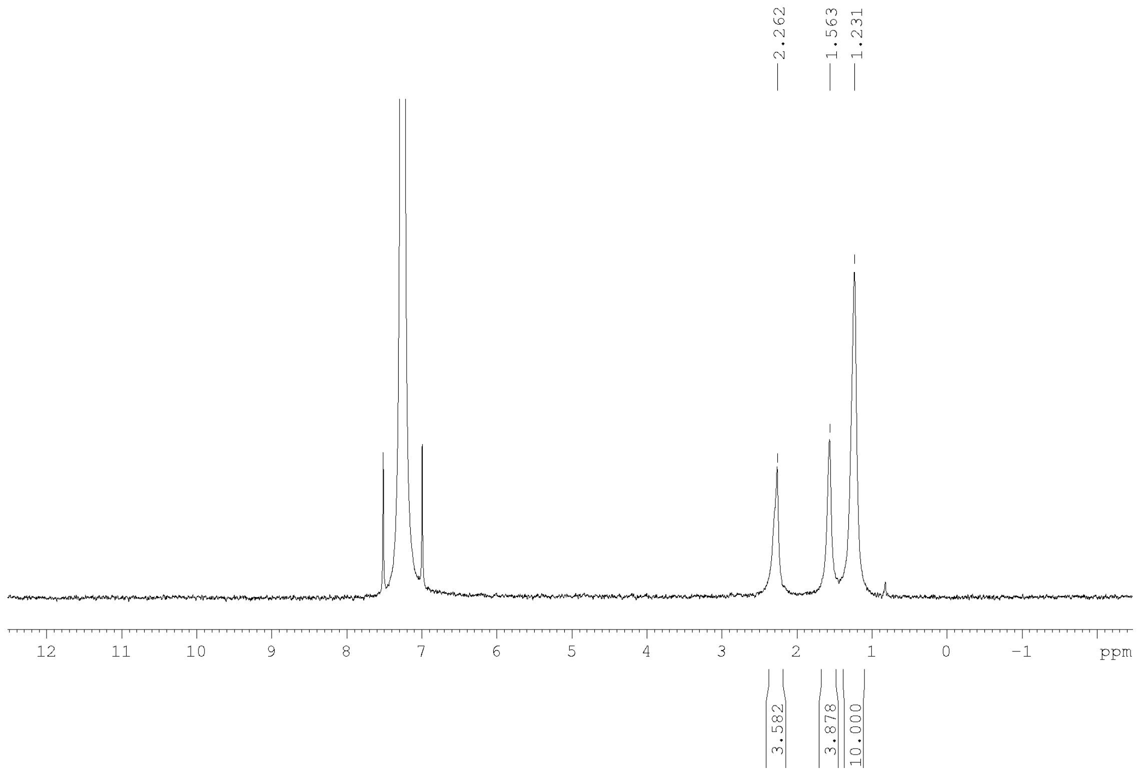

Supplementary Figure 17. ^{2}H NMR (60 MHz, $CDCl_3$) of 11-methoxy-11-oxoundecanoic acid-d_{18}

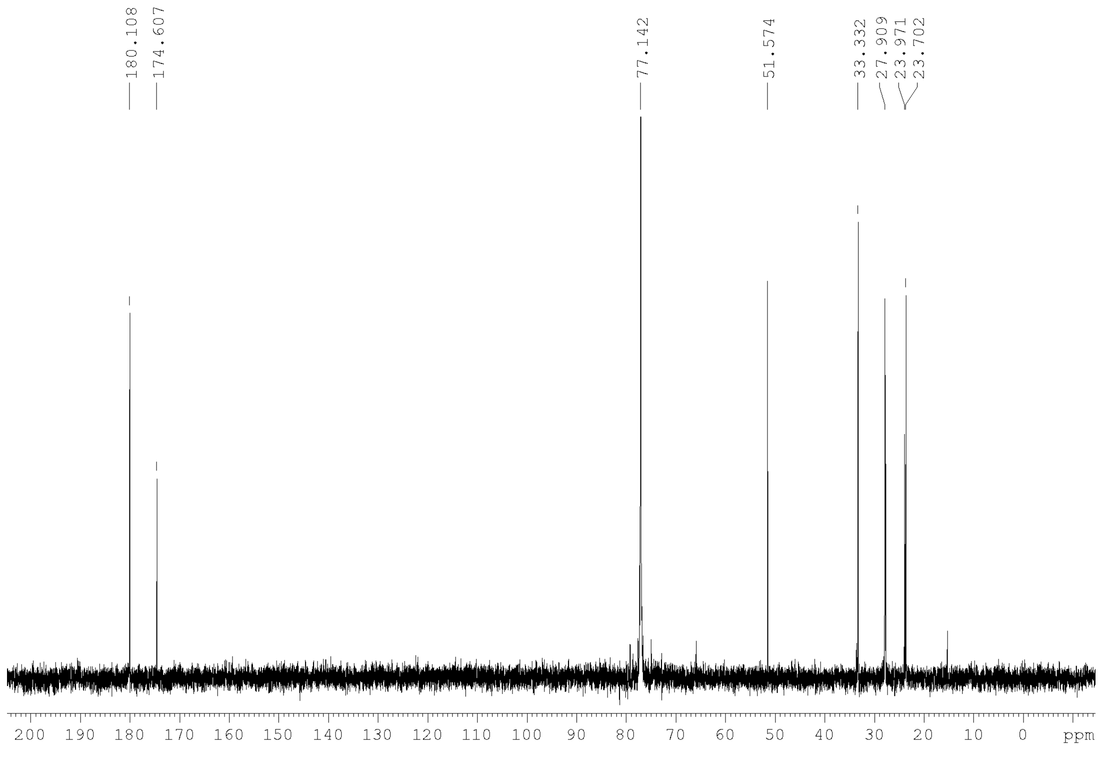

Supplementary Figure 18. ^{13}C NMR {^{1}H, ^{2}H decoupled} (100 MHz, $CDCl_3$) of 11-methoxy-11-oxoundecanoic acid-d_{18}

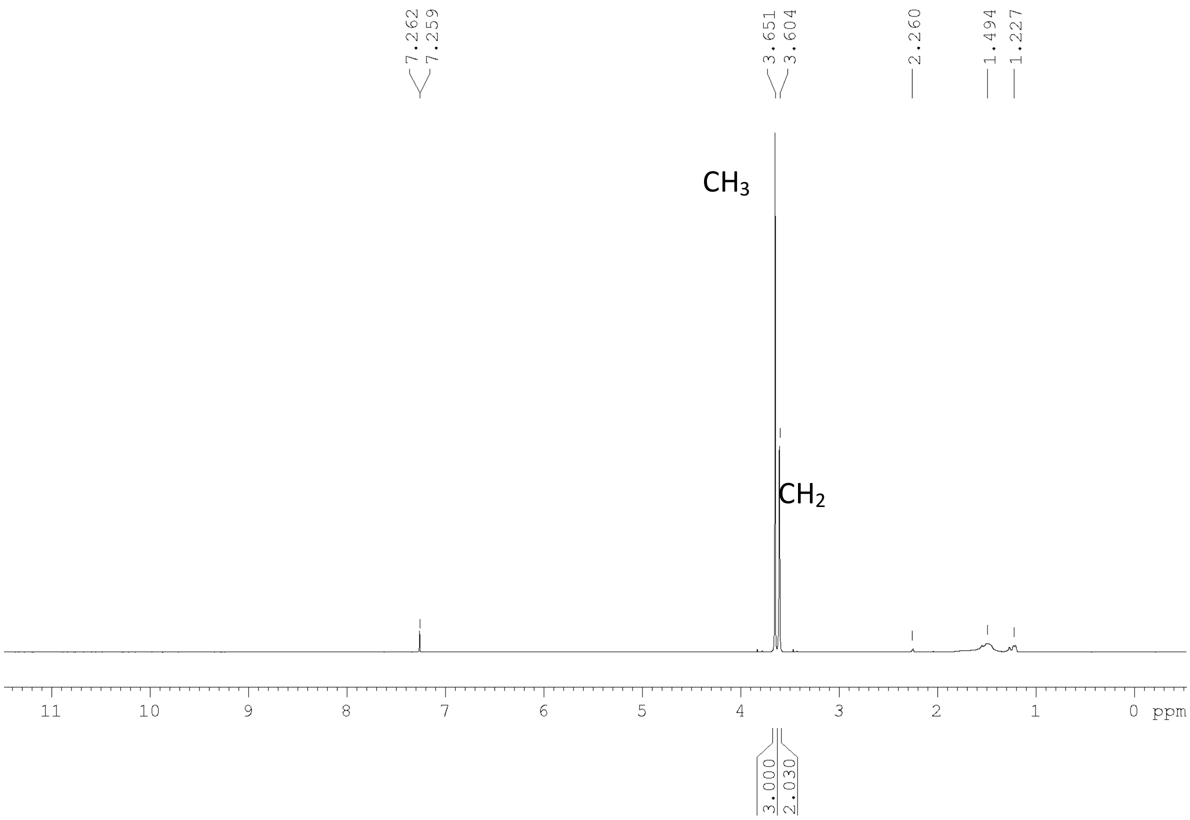

Supplementary Figure 19. ^{1}H NMR (400 MHz, $CDCl_3$) methyl 11-hydroxyundecanoate-d_{18}

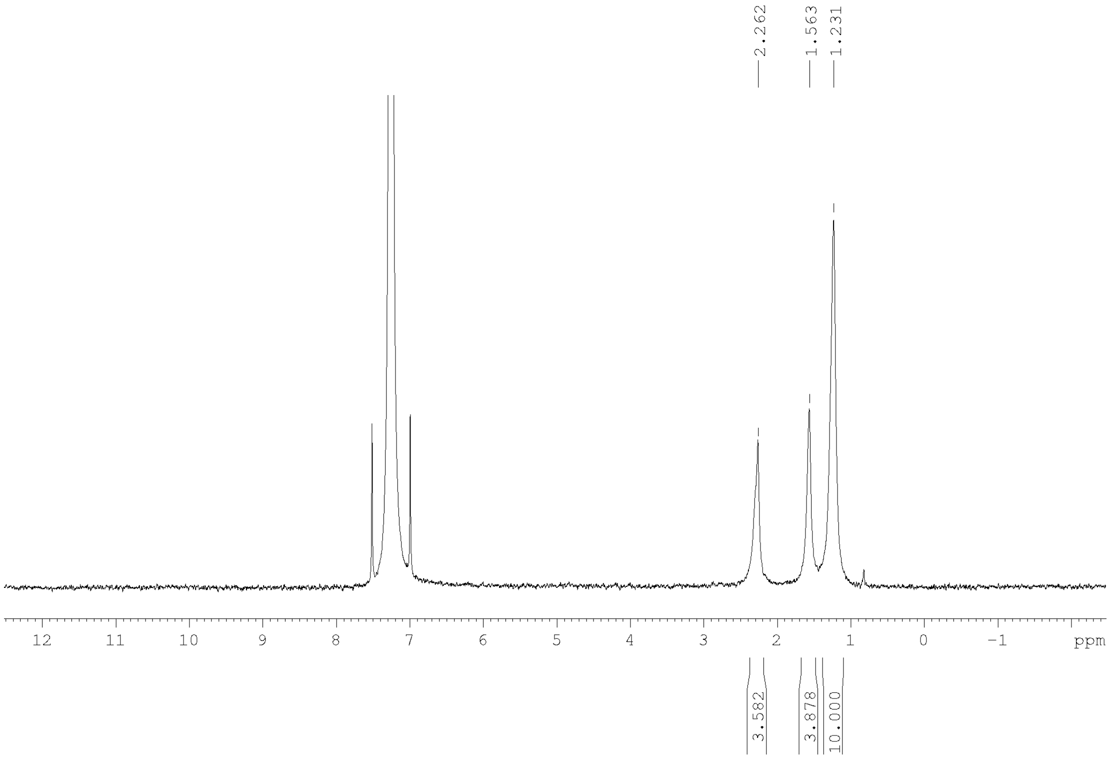

Supplementary Figure 20. ^{2}H NMR (60 MHz, $CDCl_3$) methyl 11-hydroxyundecanoate-d_{18}

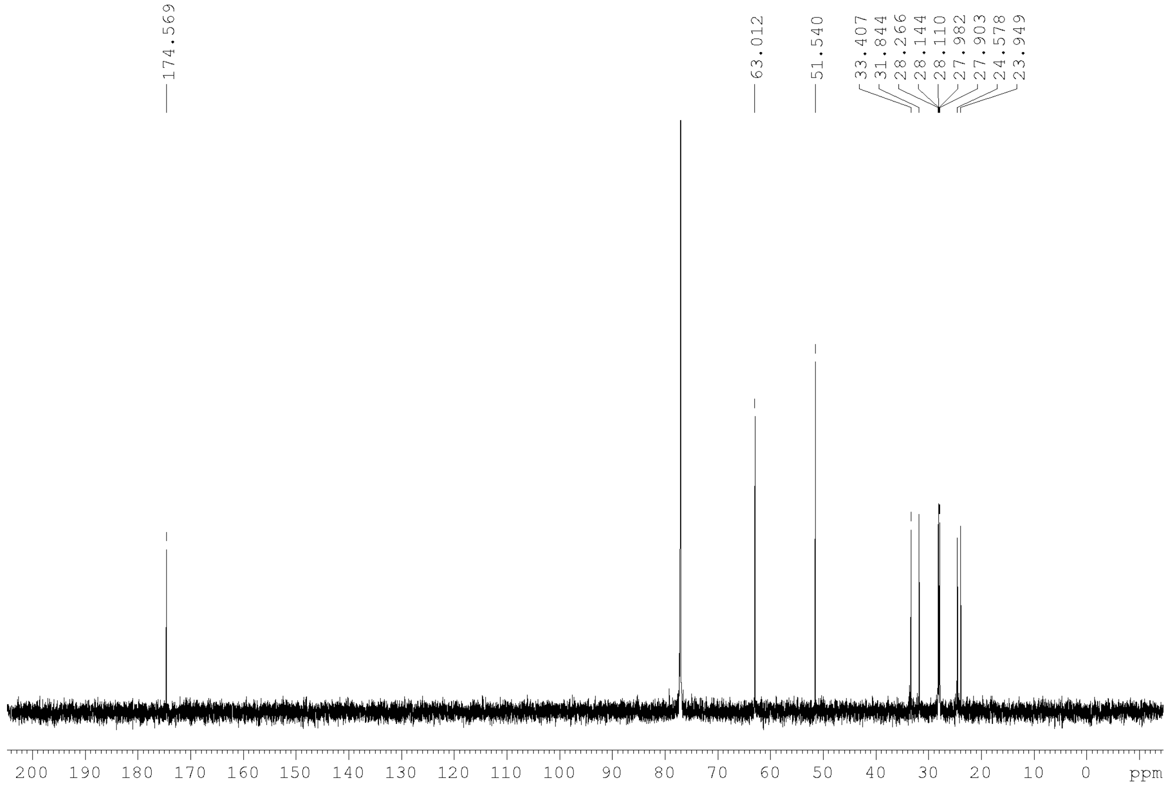

Supplementary Figure 21. ^{13}C NMR {^{1}H, ^{2}H decoupled} (100 MHz, $CDCl_3$) methyl 11-hydroxyundecanoate-d_{18}

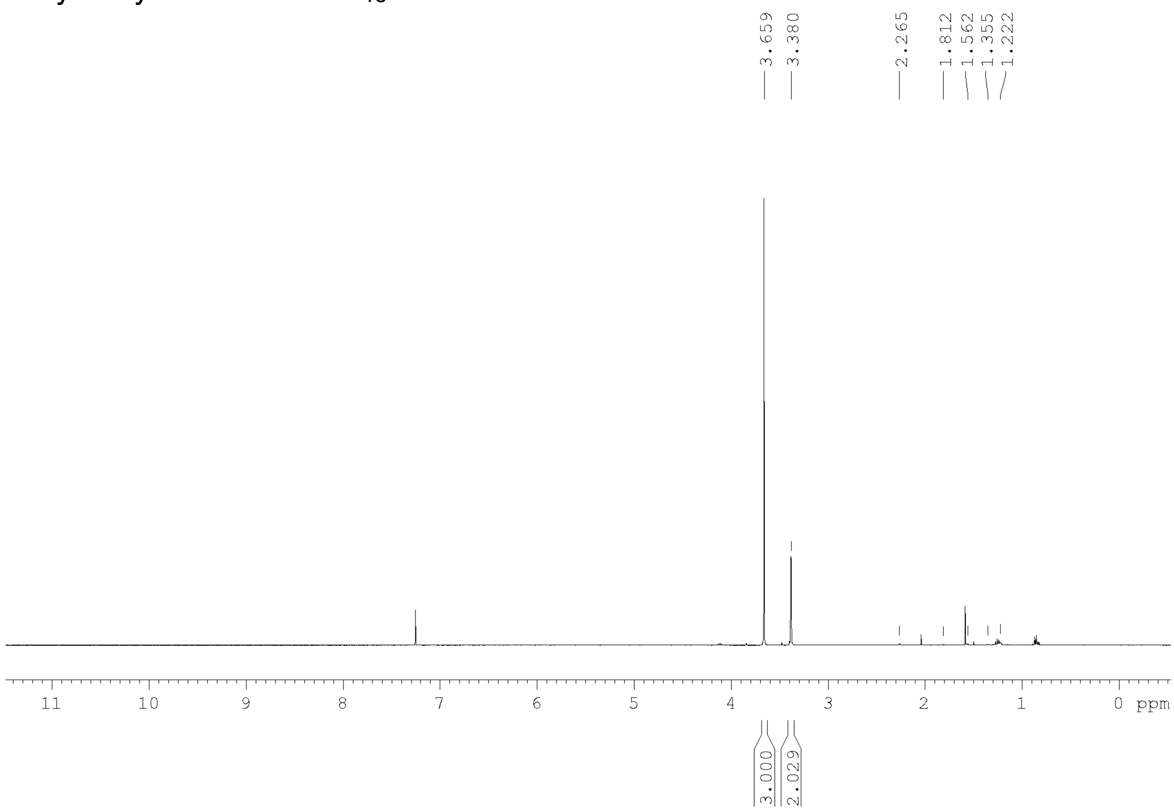

Supplementary Figure 22. ^{1}H NMR (400 MHz, $CDCl_3$) methyl 11-bromoundecanoate-d_{18}

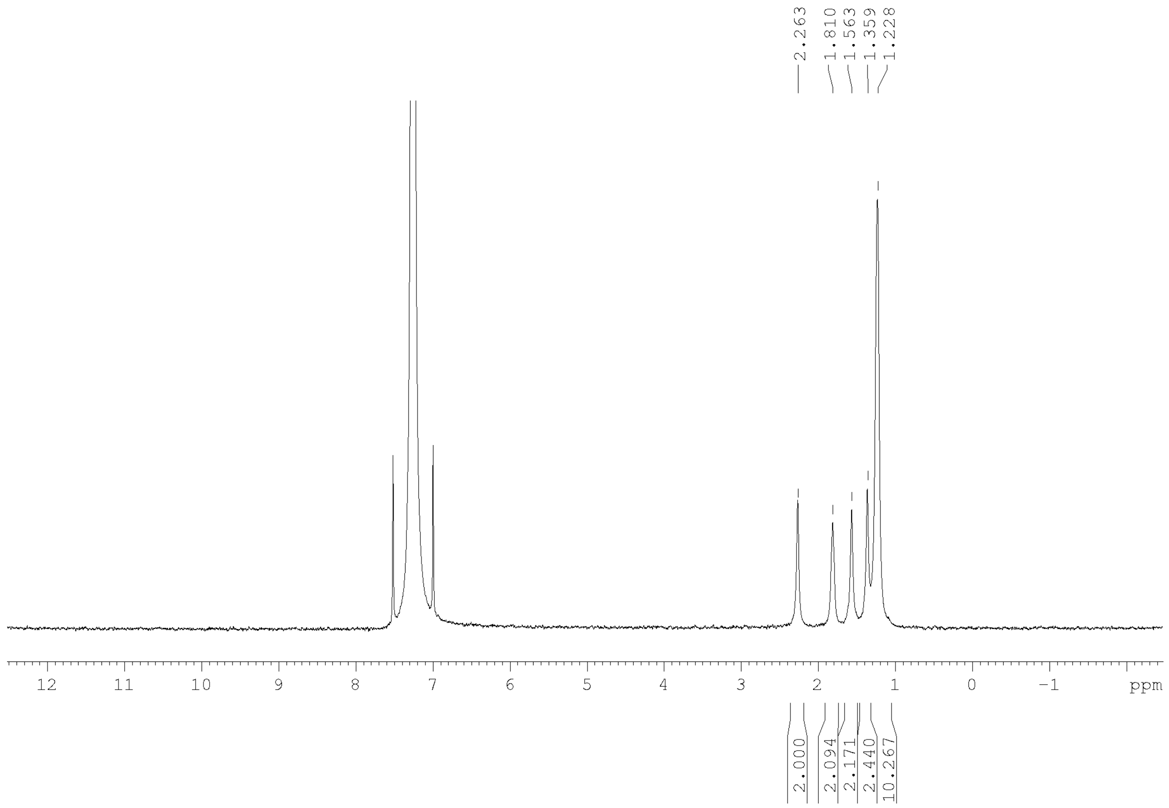

Supplementary Figure 23. ^{2}H NMR (60 MHz, $CDCl_3$) methyl 11-bromoundecanoate-*d*$_{18}$

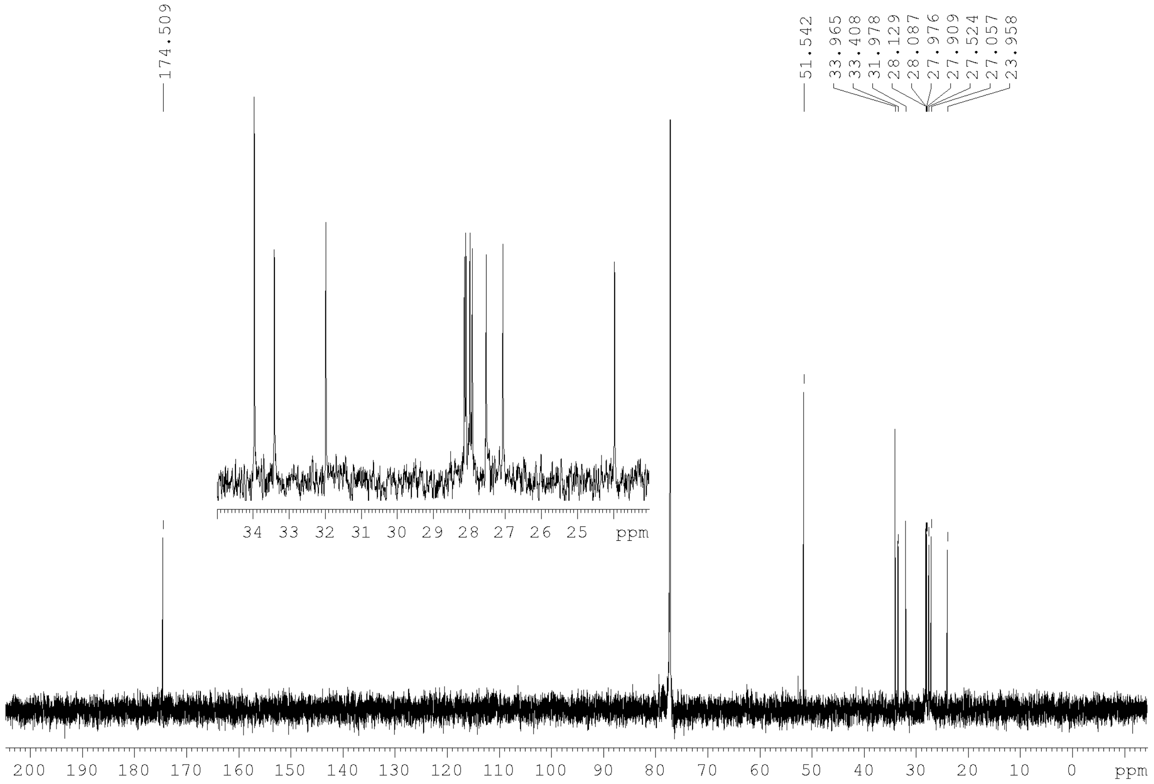

Supplementary Figure 24. ^{13}C NMR {^{1}H, ^{2}H decoupled} (100 MHz, $CDCl_3$) methyl 11-bromoundecanoate-*d*$_{18}$

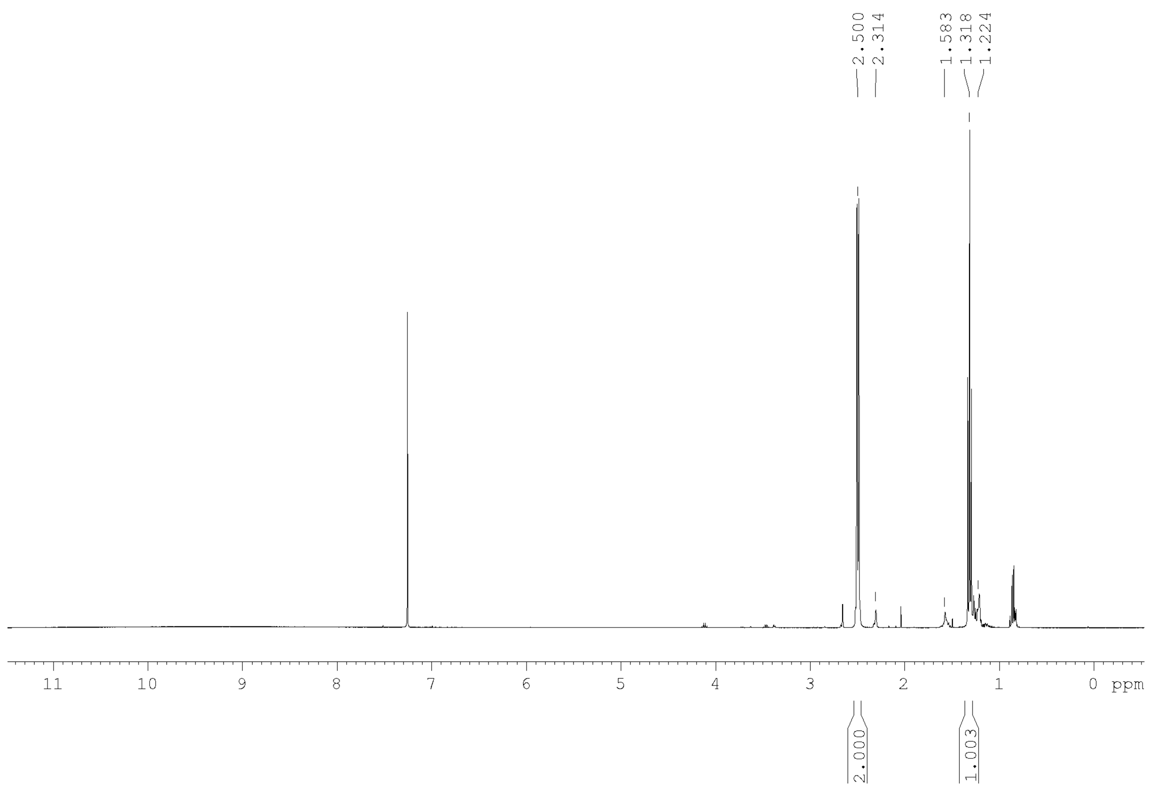

Supplementary Figure 25. ^{1}H NMR (400 MHz, $CDCl_3$) 11-mercaptoundecanoic acid-d_{18}

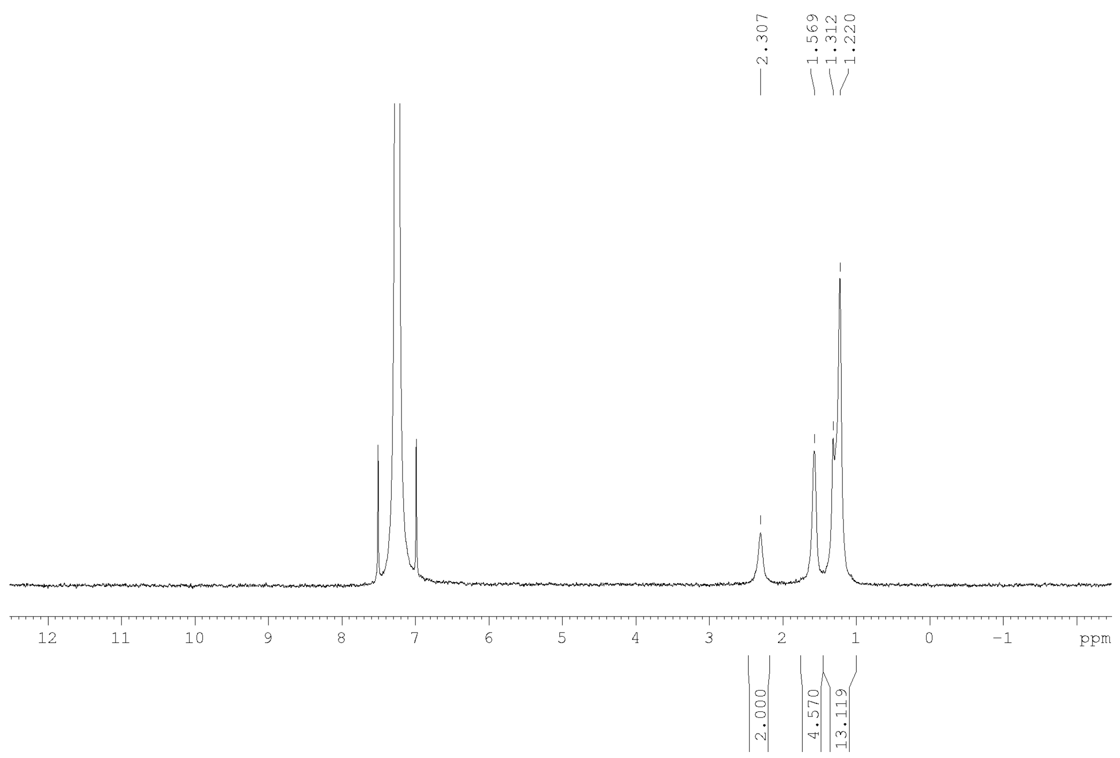

Supplementary Figure 26. ^{2}H NMR (60 MHz, $CDCl_3$) 11-mercaptoundecanoic acid-d_{18}

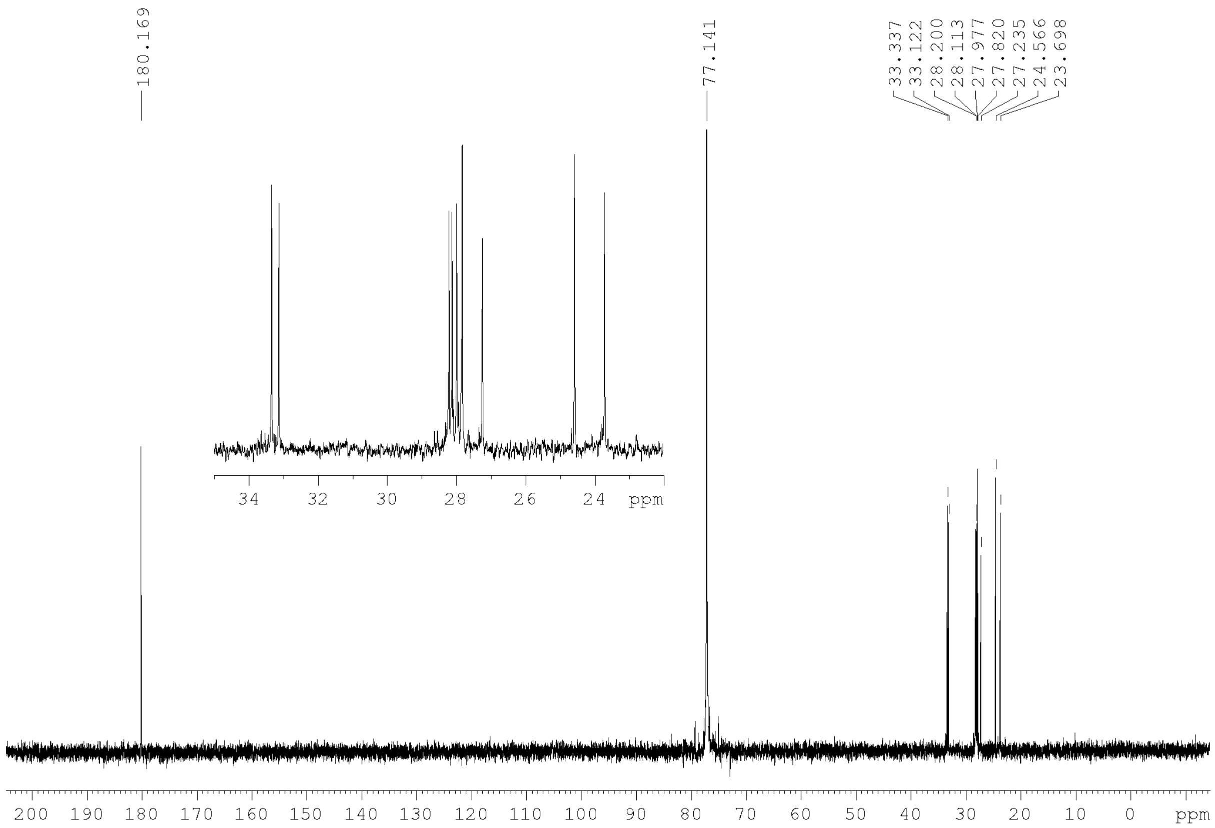

Supplementary Figure 27. ^{13}C NMR {^{1}H, ^{2}H decoupled} (100 MHz, $CDCl_3$) 11-mercaptoundecanoic acid-d_{18}

Acq. File: 20140611_AMK05057.wiff Sample Name: AMK05057 ER DP=-90V
Sample Number: N/A

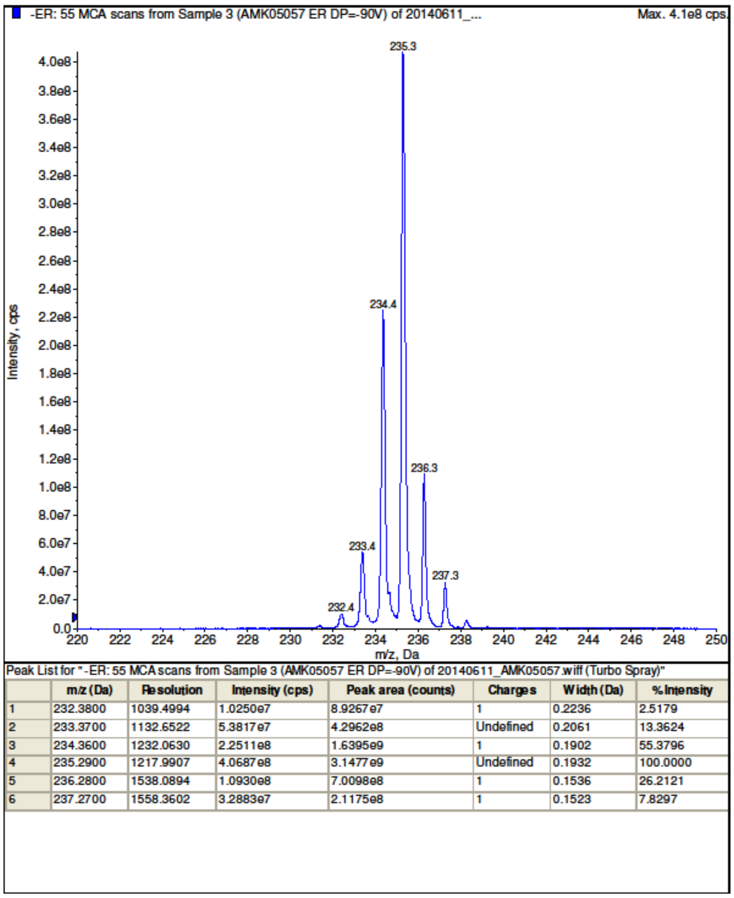

Peak List for "-ER: 55 MCA scans from Sample 3 (AMK05057 ER DP=-90V) of 20140611_AMK05057.wiff (Turbo Spray)"

	m/z (Da)	Resolution	Intensity (cps)	Peak area (counts)	Charges	Width (Da)	% Intensity
1	232.3800	1039.4994	1.0250e7	8.9267e7	1	0.2236	2.5179
2	233.3700	1132.6522	5.3817e7	4.2962e8	Undefined	0.2061	13.3624
3	234.3600	1232.0630	2.2511e8	1.6395e9	1	0.1902	55.3796
4	235.2900	1217.9907	4.0687e8	3.1477e9	Undefined	0.1932	100.0000
5	236.2800	1538.0894	1.0930e8	7.0098e8	1	0.1536	26.2121
6	237.2700	1558.3602	3.2883e7	2.1175e8	1	0.1523	7.8297

Supplementary Figure 28. Enhanced resolution (ER) ESI-MS spectrum of 11-mercaptoundecanoic acid (-90V).

Calculation of % Deuteration from MS Isotope Distribution

Note: Always label the lowest m/z peak as Peak No. 1 & increment each subsequent peak by 1 m/z unit

Parent Ion Peak No.	Theoretical % D for Peak	Peak Area	Corrected Peak Area	Contribution of Peak to Overall % D
1	5.0	0.0000E+00	0.0000E+00	0.0
2	10.0	0.0000E+00	0.0000E+00	0.0
3	15.0	0.0000E+00	0.0000E+00	0.0
4	20.0	0.0000E+00	0.0000E+00	0.0
5	25.0	0.0000E+00	0.0000E+00	0.0
6	30.0	0.0000E+00	0.0000E+00	0.0
7	35.0	0.0000E+00	0.0000E+00	0.0
8	40.0	0.0000E+00	0.0000E+00	0.0
9	45.0	0.0000E+00	0.0000E+00	0.0
10	50.0	0.0000E+00	0.0000E+00	0.0
11	55.0	0.0000E+00	0.0000E+00	0.0
12	60.0	0.0000E+00	0.0000E+00	0.0
13	65.0	0.0000E+00	0.0000E+00	0.0
14	70.0	0.0000E+00	0.0000E+00	0.0
15	75.0	8.9267E+07	8.9267E+07	1.2
16	80.0	4.2962E+08	4.1882E+08	6.3
17	85.0	1.6395E+09	1.6395E+09	26.0
18	90.0	3.1477E+09	2.9493E+09	49.5
19	95.0	7.0098E+08	2.5617E+08	4.5
20	100.0	2.1175E+08	4.1711E+06	0.1

^{13}C Isotopic Distribution from ChemDraw:	
^{13}C # 2	12.1
^{13}C # 3	3.9

Total Corrected Area of all Isotopic Peaks
5.3572E+09

Average % Deuteration of
**87.7**

Error = +/- 2 %

Theoretical % D for Peak	Corrected Peak Area	Amount (%) of compound at Theoretical % D
5.0	0.0000E+00	0.0
10.0	0.0000E+00	0.0
15.0	0.0000E+00	0.0
20.0	0.0000E+00	0.0
25.0	0.0000E+00	0.0
30.0	0.0000E+00	0.0
35.0	0.0000E+00	0.0
40.0	0.0000E+00	0.0
45.0	0.0000E+00	0.0
50.0	0.0000E+00	0.0
55.0	0.0000E+00	0.0
60.0	0.0000E+00	0.0
65.0	0.0000E+00	0.0
70.0	0.0000E+00	0.0
75.0	8.9267E+07	1.7
80.0	4.1882E+08	7.8
85.0	1.6395E+09	30.6
90.0	2.9493E+09	55.1
95.0	2.5617E+08	4.8
100.0	4.1711E+06	0.1

Supplementary Figure 29. Calculation of overall deuterium content from mass spec data presented above.

Synthesis of phenylethanethiol-d_7

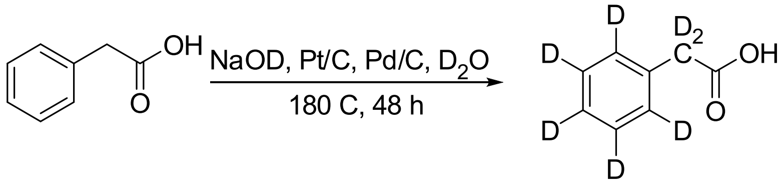

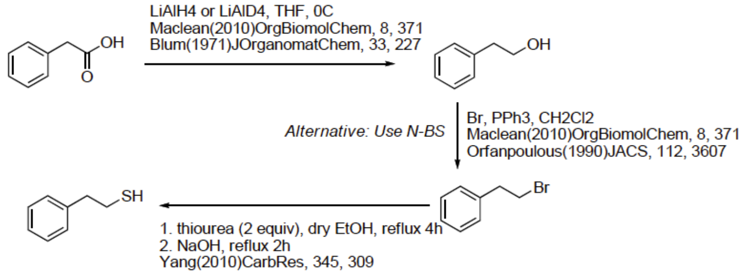

Phenylacetic acid (10 g, 73.5 mmol), NaOD solution (40% w/w, 5.2 mL, 1 equiv.), Pt/C (10% w/w, 1.0 g), Pd/C (10% w/w, 1.0 g) and D_2O (120 mL) were purged with $N_{2(g)}$ and then $H_{2(g)}$ stirred under hydrothermal conditions for 48 hours at 180 °C in a Mini Benchtop 4560 Parr reactor (600 mL vessel capacity, 206 bar max. pressure, 350 °C max. temperature). The vessel was cooled, and the reaction mixture filtered over Celite and washed with water to remove the catalyst. The filtrate was acidified with 1 M HCl, and extracted with ethyl acetate, dried over Na_2SO_4 and evaporated to give a white solid that was recrystallised from boiling H_2O (5.9 g, 55%).

2.1 g Phenyl acetic acid was dissolved in 40ml dry THF under ice bath and then dropwise added to the solution of 1.5 g lithium aluminium hydride in 30 ml dry THF. The reaction mixture was stirred at room temperature overnight. The reaction was quenched by adding HCl and most of the THF was evaporated. The mixture was dissolved in EtOAc and washed with H2O and brine, dried (1.8g, 90%).

1.8 g phenylethanol was dissolved in 20ml dry DCM and was added dropwise 10ml DCM solution of 1.61ml PBr3. The reaction mixture was allowed to stir for 3 hours and quenched with 5ml M-Q water and then washed three times with brine, NaHCO3 solution and H2O. The product was dried with rotavap at 60 degrees, yielding 1.47 g 2-Phenylethyl bromide (81%).

1.47g 2-Phenylethyl bromide was dissolved in 20 ml dry ethanol and 3g of thiourea was added to solution under stirring. The reaction proceeded overnight under reflux. 10 ml 1M NaOH solution was then added and reaction further for 4 hours. The reaction mixture was neutralized with 0.1M HCl solution and then extracted and washed with DCM. The crude product was purified by silica gel column chromatography (EtOAC: Hex= 2:1) to give 1.1 g of phenylethanethiol (74%).

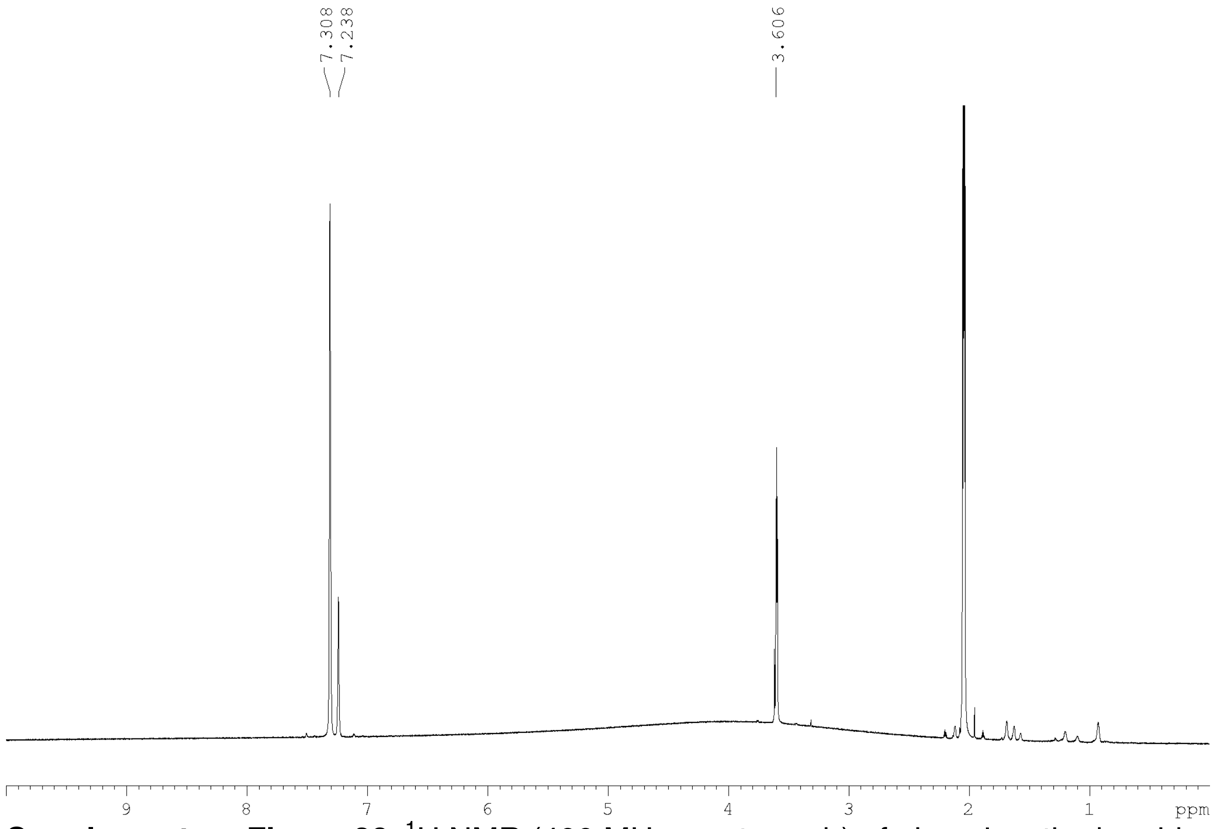

Supplementary Figure 30. ^{1}H NMR (400 MHz, acetone-d_6) of phenylacetic-d_7 acid

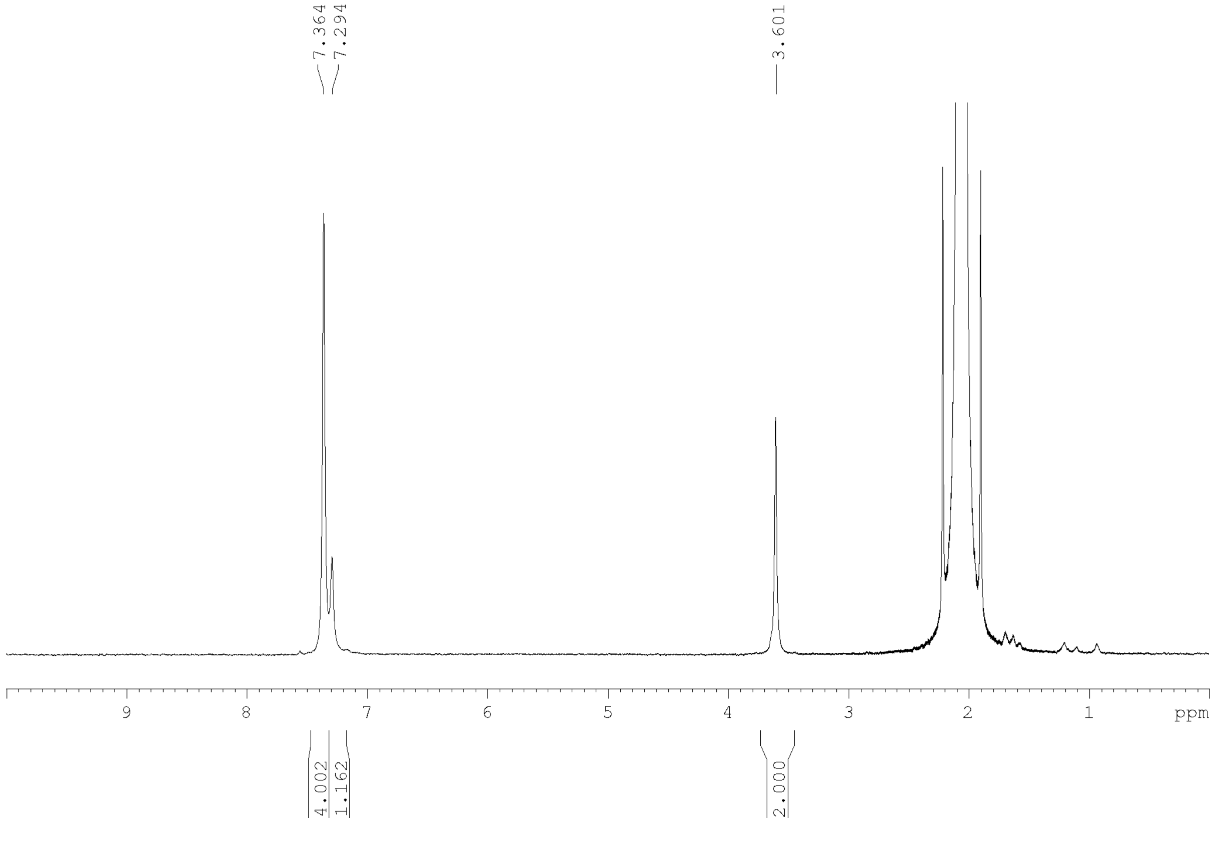

Supplementary Figure 31. ^{2}H NMR (60 MHz, acetone-d_6) of phenylacetic-d_7 acid

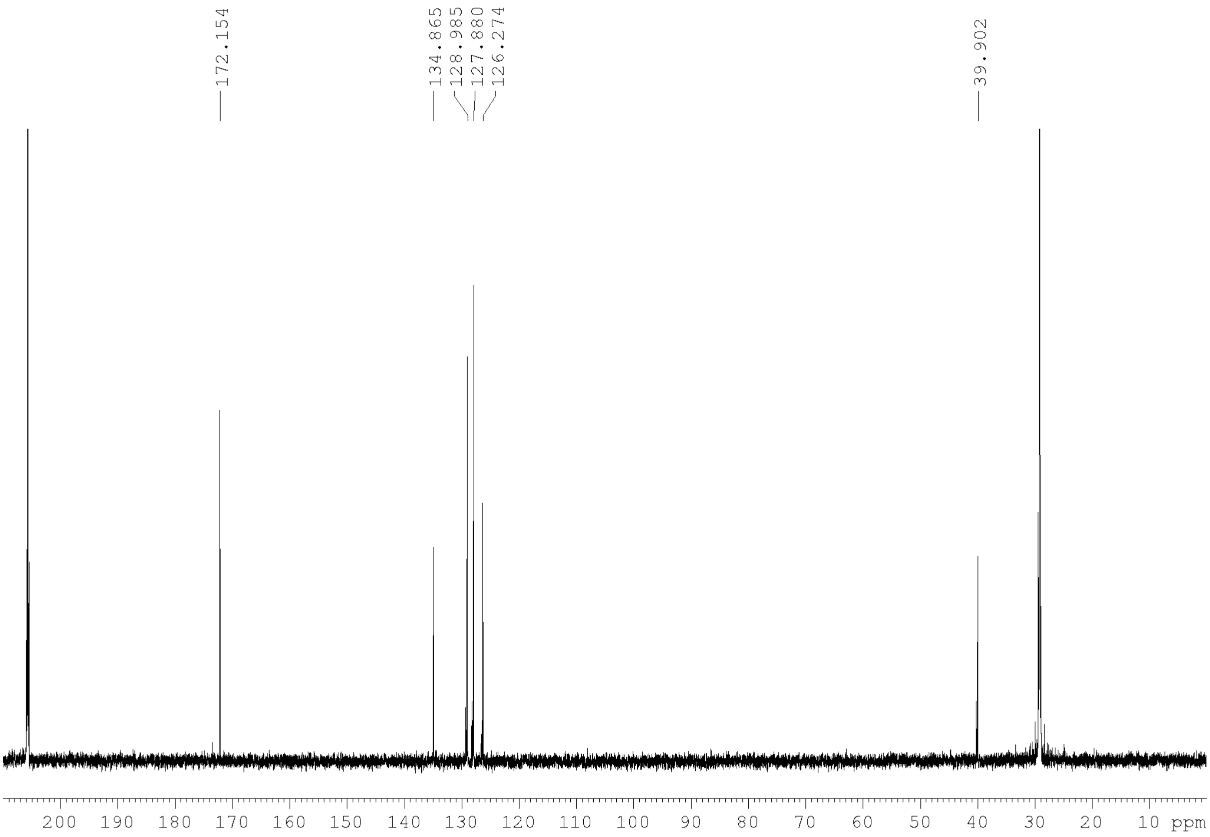

Supplementary Figure 32. ^{13}C NMR {^{1}H, ^{2}H decoupled} (100 MHz, acetone-d_6) of phenylacetic-d_7 acid

Acq. File: 20140619_AMK05059.wiff

Sample Name: AMK05059, ER, DP=-20V
Sample Number: N/A

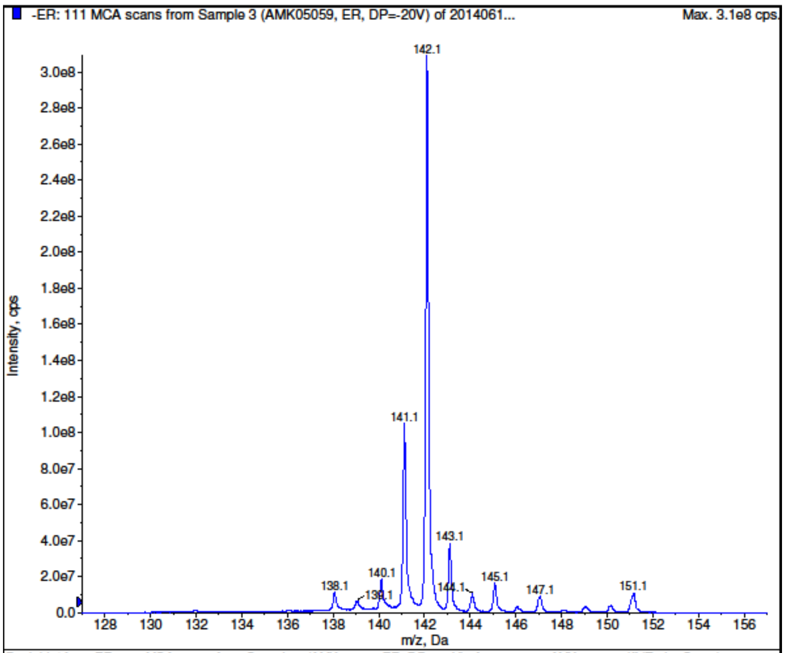

Peak List for "-ER: 111 MCA scans from Sample 3 (AMK05059, ER, DP=-20V) of 20140619_AMK05059.wiff (Turbo Spray)"

	m/z (Da)	Resolution	Intensity (cps)	Peak area (counts)	Charges	Width (Da)	% Intensity
1	138.0600	835.0701	1.1517e7	7.4200e7	Undefined	0.1653	3.7959
2	139.0800	537.9119	6.4333e6	6.1867e7	1	0.2585	2.1257
3	140.1300	853.1299	1.8433e7	1.2270e8	Undefined	0.1642	6.0451
4	141.1200	1029.3502	1.0527e8	6.9230e8	1	0.1371	33.9812
5	142.1100	1093.5264	3.0884e8	1.7520e9	Undefined	0.1300	100.0000
6	143.1000	1059.8418	3.8683e7	2.3108e8	1	0.1350	12.4900
7	144.0900	863.3699	1.0542e7	7.3650e7	1	0.1669	3.4816
8	145.0800	906.9161	1.6525e7	1.0855e8	Undefined	0.1600	5.4369
9	147.0600	733.3137	9.1083e6	7.2350e7	Undefined	0.2005	3.1315
10	151.1400	660.3190	1.0908e7	9.1742e7	Undefined	0.2289	3.6928

Supplementary Figure 33. Enhanced resolution (ER) mass spectrum of phenylacetic-d_7 acid.

Calculation of % Deuteration from MS Isotope Distribution

Note: Always label the lowest m/z peak as Peak No. 1 & increment each subsequent peak by 1 m/z unit

Parent Ion Peak No.	Theoretical % D for Peak	Peak Area	Corrected Peak Area	Contribution of Peak to Overall % D
1	0.0	0.0000E+00	0.0000E+00	0.0
2	14.3	0.0000E+00	0.0000E+00	0.0
3	28.6	0.0000E+00	0.0000E+00	0.0
4	42.9	7.4200E+07	7.4200E+07	1.3
5	57.1	6.1800E+07	6.1800E+07	1.4
6	71.4	1.2270E+08	1.0910E+08	3.1
7	85.7	6.9230E+08	6.6531E+08	22.7
8	100.0	1.7520E+09	1.5997E+09	63.7
9				0.0
10				0.0
11				0.0
12				0.0
13				0.0
14			0.0000E+00	0.0
15			0.0000E+00	0.0
16			0.0000E+00	0.0
17			0.0000E+00	0.0

^{13}C Isotopic Distribution from ChemDraw:

^{13}C # 2	22
^{13}C # 3	

Total Corrected Area of all Isotopic Peaks

2.5101E+09

Average % Deuteration of

92.2

Error = +/- 2 %

Theoretical % D for Peak	Corrected Peak Area	Amount (%) of compound at Theoretical % D
0.0	0.0000E+00	0.0
14.3	0.0000E+00	0.0
28.6	0.0000E+00	0.0
42.9	7.4200E+07	3.0
57.1	6.1800E+07	2.5
71.4	1.0910E+08	4.3
85.7 100.0	6.6531E+08	26.5
	1.5997E+09	63.7
0.0	0.0000E+00	0.0
0.0	0.0000E+00	0.0
0.0	0.0000E+00	0.0
0.0	0.0000E+00	0.0
0.0	0.0000E+00	0.0
0.0	0.0000E+00	0.0
0.0	0.0000E+00	0.0
0.0	0.0000E+00	0.0
0.0	0.0000E+00	0.0

Supplementary Figure 34. Calculation of percentage deuteration of phenylacetic-d_7 acid

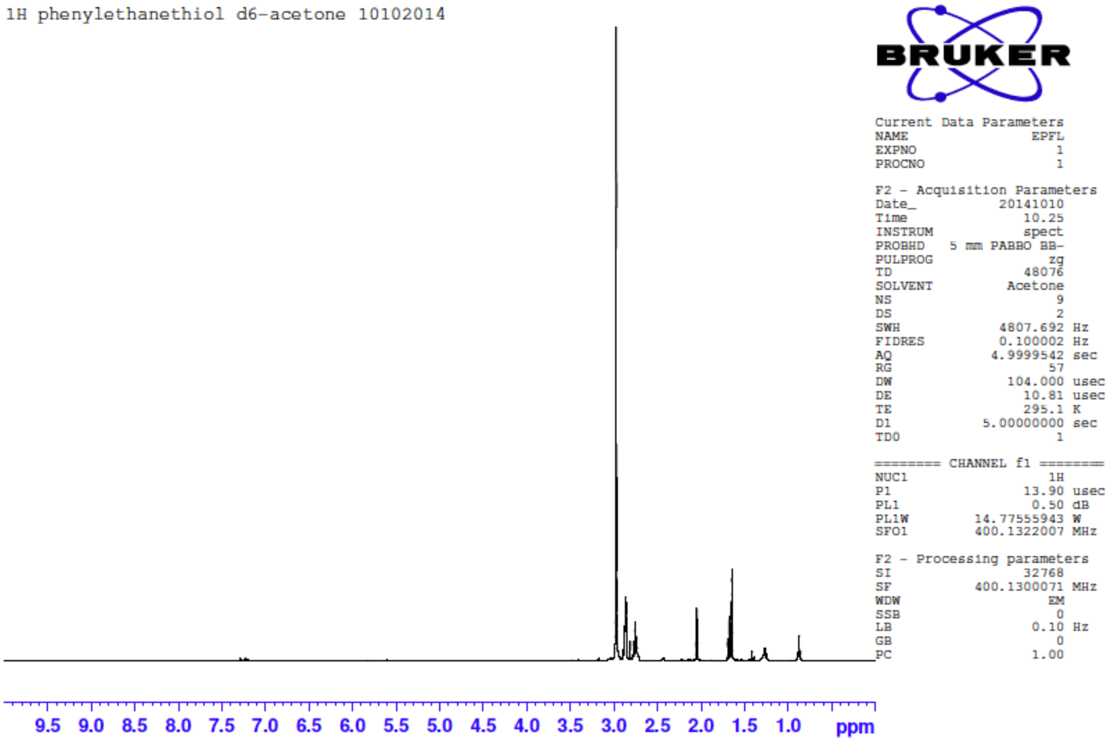

Supplementary Figure 35. ^{1}H NMR (400 MHz, acetone-d_6) of phenylethanethiol-d9

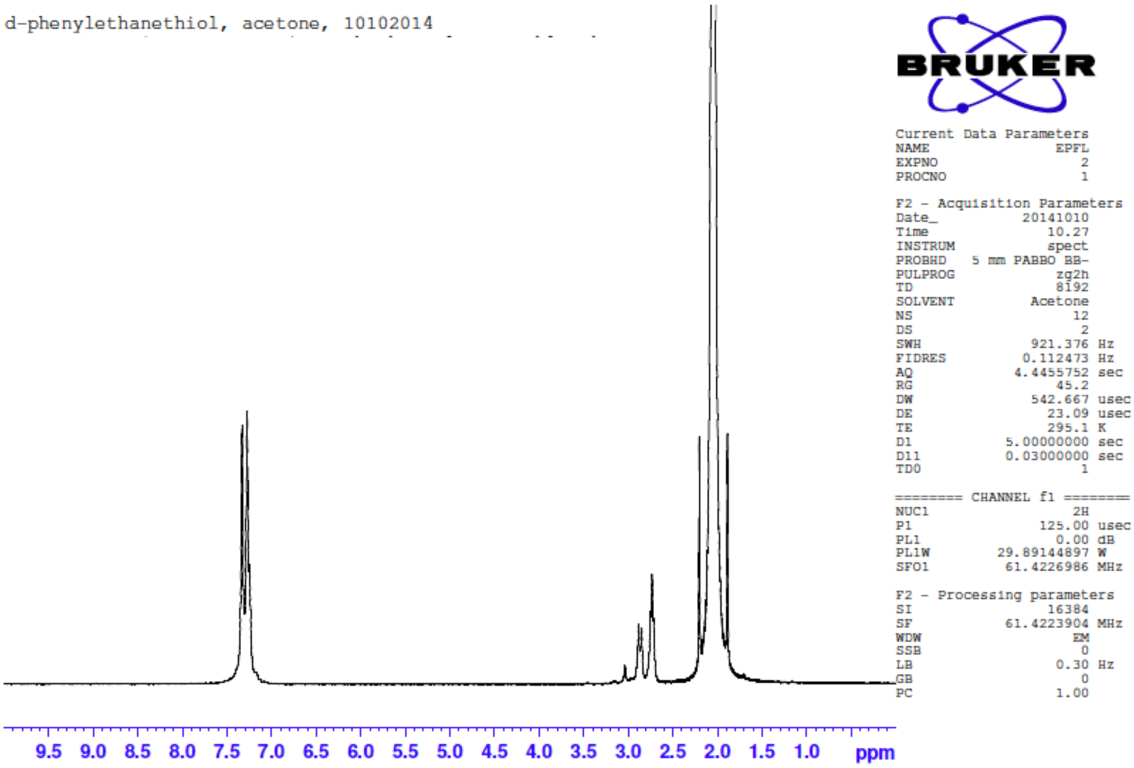

Supplementary Figure 36. ^{2}H NMR (60 MHz, acetone-d_6) of phenylethanethiol-d9

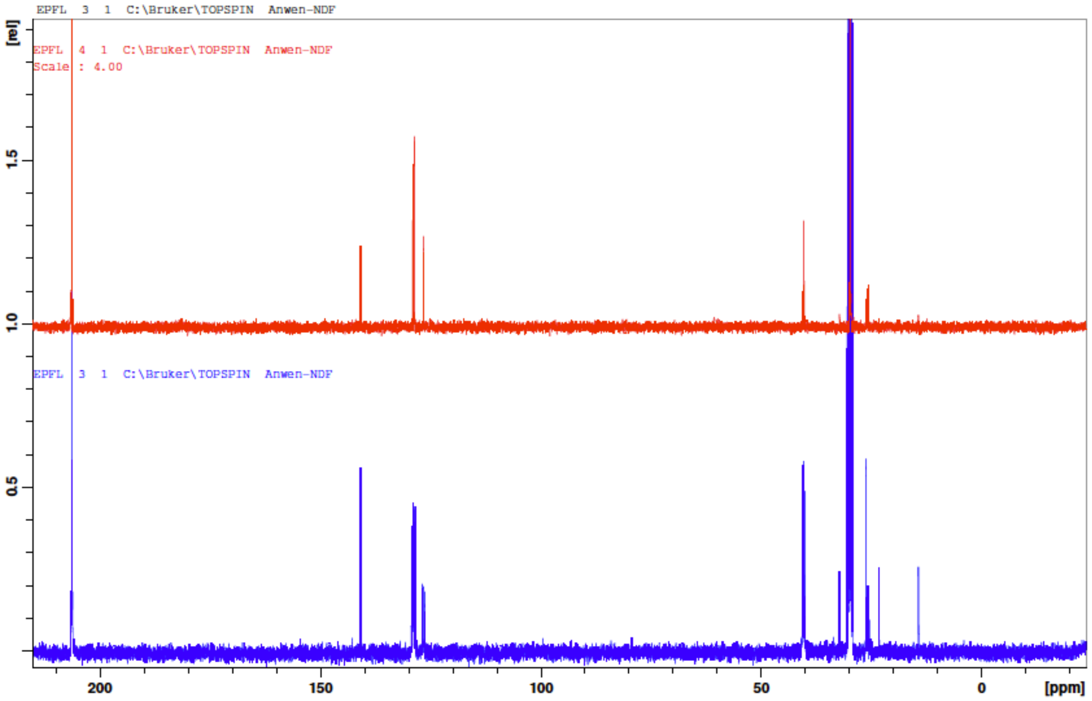

Supplementary Figure 37. ^{13}C NMR {^{1}H, ^{2}H decoupled} (100 MHz, acetone-d_6) of phenylethanethiol-d9

References

1 Groot, R. D. & Warren, P. B. Dissipative particle dynamics: Bridging the gap between atomistic and mesoscopic simulation. *J. Chem. Phys.* **107**, 4423-4435, (1997).

2 Posel, Z., Posocco, P., Lisal, M., Fermeglia, M. & Pricl, S. Highly grafted polystyrene/polyvinylpyridine polymer gold nanoparticles in a good solvent: Effects of chain length and composition. *Soft Matter* **12**, 3600-3611, (2016).

3 Langner, K. M. & Sevink, G. J. A. Mesoscale modeling of block copolymer nanocomposites. *Soft Matter* **8**, 5102-5118, (2012).

4 Walker, M., Masters, A. J. & Wilson, M. R. Self-assembly and mesophase formation in a non-ionic chromonic liquid crystal system: Insights from dissipative particle dynamics simulations. *Phys. Chem. Chem. Phys.* **16**, 23074-23081, (2014).

5 Posocco, P. *et al.* Interfacial tension of oil/water emulsions with mixed non-ionic surfactants: Comparison between experiments and molecular simulations. *RSC Advances* **6**, 4723-4729, (2016).

6 Vishnyakov, A., Lee, M.-T. & Neimark, A. V. Prediction of the critical micelle concentration of nonionic surfactants by dissipative particle dynamics simulations. *J. Phys. Chem. Lett.* **4**, 797-802, (2013).

7 Suttipong, M., Grady, B. P. & Striolo, A. Surfactant aggregates templated by lateral confinement. *J. Phys. Chem. B* **119**, 5467-5474, (2015).

8 Berezkin, A. V. & Kudryavtsev, Y. V. Simulation of end-coupling reactions at a polymer−polymer interface: The mechanism of interfacial roughness development. *Macromolecules* **44**, 112-121, (2011).
9 Singh, C. *et al.* Entropy-mediated patterning of surfactant-coated nanoparticles and surfaces. *Phys. Rev. Lett.* **99**, (2007).
10 Pons-Siepermann, I. C. & Glotzer, S. C. Design of patchy particles using quaternary self-assembled monolayers. *ACS Nano* **6**, 3919–3924, (2012).
11 Posocco, P. *et al.* Self-organization of mixtures of fluorocarbon and hydrocarbon amphiphilic thiolates on the surface of gold nanoparticles. *ACS Nano* **6**, 7243-7253, (2012).
12 Plimpton, S. Fast parallel algorithms for short-range molecular dynamics. *J. Comput. Phys.* **117**, 1-19, (1995).
13 Brown, W. M., Kohlmeyer, A., Plimpton, S. J. & Tharrington, A. N. Implementing molecular dynamics on hybrid high performance computers – particle–particle particle-mesh. *Comput. Phys. Commun.* **183**, 449-459, (2012).
14 Nguyen, T. D. & Plimpton, S. J. Accelerating dissipative particle dynamics simulations for soft matter systems. *Comput. Mater. Sci.* **100**, Part B, 173-180, (2015).
15 Gezelter, J. D. *et al. OPENMD, an open source engine for molecular dynamics.* Available at http://openmd.net.
16 Scocchi, G. *et al.* A complete multiscale modelling approach for polymer–clay nanocomposites. *Chem. Eur. J.* **15**, 7586-7592, (2009).
17 Martínez, L., Andrade, R., Birgin, E. G. & Martnez, J. M. Packmol: A package for building initial configurations for molecular dynamics simulations. *J. Comput. Chem.* **30**, 2157−2164, (2009).
18 Scocchi, G., Posocco, P., Fermeglia, M. & Pricl, S. Polymer−clay nanocomposites: A multiscale molecular modeling approach. *J. Phys. Chem. B* **111**, 2143-2151, (2007).
19 Scocchi, G., Posocco, P., Danani, A., Pricl, S. & Fermeglia, M. To the nanoscale, and beyond! Multiscale molecular modeling of polymer-clay nanocomposites. *Fluid Phase Equilib.* **261**, 366-374, (2007).
20 Toth, R. *et al.* Multiscale computer simulation studies of water-based montmorillonite/poly(ethylene oxide) nanocomposites. *Macromolecules* **42**, 8260-8270, (2009).
21 Şologan, M. *et al.* Patchy and janus nanoparticles by self-organization of mixtures of fluorinated and hydrogenated alkanethiolates on the surface of a gold core. *ACS Nano* **10**, 9316-9325, (2016).
22 Heinz, H., Lin, T.-J., Kishore Mishra, R. & Emami, F. S. Thermodynamically consistent force fields for the assembly of inorganic, organic, and biological nanostructures: The interface force field. *Langmuir* **29**, 1754-1765, (2013).
23 Goel, H., Chandran, P. R., Mitra, K., Majumdar, S. & Ray, P. Estimation of interfacial tension for immiscible and partially miscible liquid systems by dissipative particle dynamics. *Chem. Phys. Lett.* **600**, 62-67, (2014).
24 Humphrey, W., Dalke, A. & Schulten, K. VMD: Visual molecular dynamics. *J. Mol. Graphics* **14**, 33-38, (1996).
